# Truvari: refined structural variant comparison preserves allelic diversity

**DOI:** 10.1186/s13059-022-02840-6

**Published:** 2022-12-27

**Authors:** Adam C. English, Vipin K. Menon, Richard A. Gibbs, Ginger A. Metcalf, Fritz J. Sedlazeck

**Affiliations:** Baylor College of Medicine Human Genome Sequencing Center, Houston, TX USA

**Keywords:** Structural variation, SV comparison, SV merging, SV benchmarking, SV annotation

## Abstract

The fundamental challenge of multi-sample structural variant (SV) analysis such as merging and benchmarking is identifying when two SVs are the same. Common approaches for comparing SVs were developed alongside technologies which produce ill-defined boundaries. As SV detection becomes more exact, algorithms to preserve this refined signal are needed. Here, we present Truvari—an SV comparison, annotation, and analysis toolkit—and demonstrate the effect of SV comparison choices by building population-level VCFs from 36 haplotype-resolved long-read assemblies. We observe over-merging from other SV merging approaches which cause up to a 2.2× inflation of allele frequency, relative to Truvari.

**Supplementary Information:**

The online version contains supplementary material available at 10.1186/s13059-022-02840-6.

## Background

The march of progress of genomic sequencing is constant, with accelerating speed from improving technologies being applied to growing populations/cohorts leading to discoveries from increasingly harder-to-assess genomic regions. One striking area of progress over the last two decades has been in the analysis of structural variants (SVs), which include 50-bp or larger genomic alterations. While single-nucleotide variants vastly outnumber the instances of SVs, the cumulative number of bases altered by SVs is higher due to their size, resulting in a significant impact on disease development and progression [1–3].

The detection of SVs has been enhanced most notably through the advent of long-read sequencing. No longer hindered by alignment through the repetitive elements which frequently mediate SVs [4], long-read sequencing has enabled refined characterization of SVs [5]. Simultaneously, SV benchmarking standards such as that created by the Genome in a Bottle consortium (GIAB) have provided objective measurements of the quality of SV tools which has assisted both genome researchers and software developers [6]. These improvements, however, have largely focused on SV discovery and genotyping within the context of single samples [7]. When comparing SVs across multiple samples, the question of how best to identify matching SVs remains inadequately addressed.

SV comparison is a fundamental operation of benchmarking, annotation, and merging that is required to address both technical artifacts and biological differentiation. First, when SVs are called by different sequencing experiments or heterogeneous pipelines, any combination of base-calling errors [7], differences in pipeline sensitivity [5], and alignment ambiguities around repeats [8] may cause the same SV to be placed in different positions or contain different sequences. Furthermore, the methods and parameters used for the comparison of SVs to determine if they are the same genomic events impact the outcome of the analysis. If the parameters used for SV matching are too lenient, benchmarking performance is inflated, incorrect annotations are applied, or over-merging occurs and causes unique SVs to be falsely identified as shared between samples. Over-merging is particularly problematic as it can result in an apparent loss of allelic diversity and an over-estimation of allelic frequencies. Similarly, if parameters for SV comparison are too strict and matching SVs are not identified, benchmarking performance is deflated, annotations are missed, and experiments such as association analyses may become under-powered [9].

Multiple strategies for SV comparison have been proposed. For example, SVs are considered to be equal using reciprocal overlap if a proportion of their individual sizes are overlapping. This traditionally has been applied to CNV calling (e.g., array CGH) as the breakpoints are imprecise [10]. However, reciprocal overlap is not applicable to sequence-resolved insertions, which have no physical span over the reference. With more precise breakpoints, other heuristics have been postulated such as breakpoint agreement where SVs are considered matching when their breakpoints are within a certain interval (e.g., 500–1000 bp). While this method may generally be sufficient for larger SVs, it is insensitive to subtle differences of smaller SVs or those at complex loci with multiple events. The logical progression is to also take into consideration the length of the SV to improve the threshold/wobble distance allowance for the breakpoints [11–13]. However, insertions of the same length and at the same position may vary in sequence composition. Any of these approaches, in isolation, can incorrectly identify alleles as matching.

These concerns expose the need for a systematic approach to SV comparison that begins with a high-quality set of SV calls and builds from that an understanding of the impact of SV comparison choices. To accomplish this, we built Truvari, which assists SV comparison by leveraging multiple metrics to make informed comparison choices. We incorporate lessons from Truvari being a widely used and recommended benchmark tool for SVs [6]. Truvari’s comparison approach is especially relevant given the improvements in SV calling accuracy in terms of breakpoint-exact, sequence-resolved calls that are becoming commonplace, not only from long-read sequencing but also more exact short-read SV discovery algorithms [14, 15].

We take previously published data of haplotype-resolved assemblies from 36 diverse individuals and measure the intra-sample haplotype similarity of SVs using Truvari [16, 17]. We demonstrate how even high-quality pipelines can produce similar, but not identical SV representations. These results are important to understand the impact of different methodologies on population merging. We again leverage Truvari to build project-level VCFs (pVCF) and gain insights into how SV merging choices affect biologically relevant metrics such as SV count and allele frequency. We give these insights context by using Truvari with varying matching thresholds as well as comparison to other SV merging methodologies.

## Results

### Truvari description

**Fig. 1:**
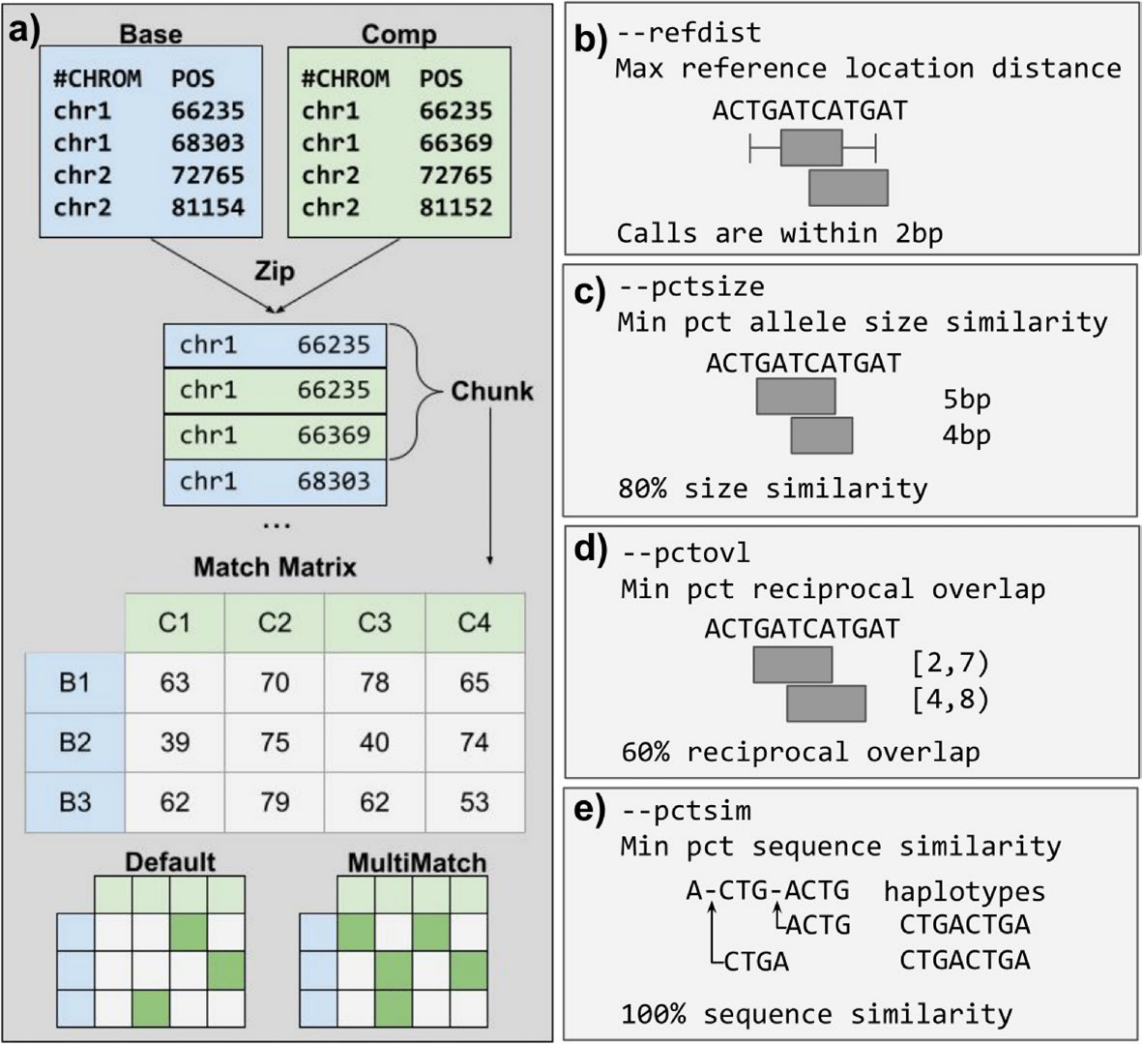
Overview of the Truvari method and comparison metrics. **a** Schematic illustrating the Truvari bench matching approach of a baseline and comparison (comp) VCF. **b**–**e** Comparison metrics used by Truvari to measure similarity

Truvari is an open-source toolkit for the comparison, annotation, and analysis of structural variation. This research focuses on the SV comparison tools for benchmarking (*bench*) and merging (*collapse*) but leverages the annotation and analysis features to enrich the information presented. Truvari’s comparison approach is detailed in the “Methods” section (Fig. 1). Briefly, Truvari compares SVs inside variant call format files (VCF) by measuring five similarity metrics between all pairs of calls within a region. These metrics are SVTYPE matching, reference distance, reciprocal overlap, size similarity, sequence similarity, and genotype matching. If any of the metrics violate user-defined thresholds, the pair of calls fails to be a candidate match.

For Truvari *bench*, default matching thresholds are set to 70% sequence and size similarity, 500 bp reference distance, svtype matching, and 0% reciprocal overlap. These thresholds were developed as part of the Genome in a Bottle consortium (GIAB) [6] and are generally applicable to most single-sample comparisons of a replicate to a ground-truth set of SVs. However, the thresholds can be raised or lowered based on the resolution of the SVs and desired stringency. For example, sequence similarity can be set to zero in order to capture matches between non-sequence-resolved calls. Truvari *collapse* default thresholds are 95% sequence and size similarity, 500-bp reference distance, identical svtype, and 0% reciprocal overlap. These default thresholds work for highly similar sequence resolved calls (e.g., calls from a harmonized pipeline) across multiple samples, but again can be tweaked to a user’s specifications.

### Matching SVs between haplotypes

To approach the central question of when to match a pair of SVs, we start with a set of 36 previously established, haplotype-resolved, long-read assemblies and call insertion and deletion SVs [16, 17]. This represents a “best case scenario” for starting with high-quality sequencing and a harmonized pipeline to minimize noise. While Truvari can process any SV type except unresolved breakends (BNDs), we focus here on only insertions and deletions. We called SVs against three references—hg19, GRCh38, and the newly published chm13—to observe how the choice of genome references impacts the calling and analysis of the SVs [18–20]. First, to ensure high accuracy of SV calling, we compared the NA24385 sample on hg19 against GIAB v0.6 Tier1 SVs using Truvari *bench* (see the “Methods” section). This measured a high precision (0.93) across each of the two haplotypes. Over 90.2% of true-positive SVs have at least 95% sequence similarity and size similarity between the generated calls and the GIAB truth set. This indicates highly consistent SV representations and that the SV calling methodology generated an accurate initial call set.

**Fig. 2:**
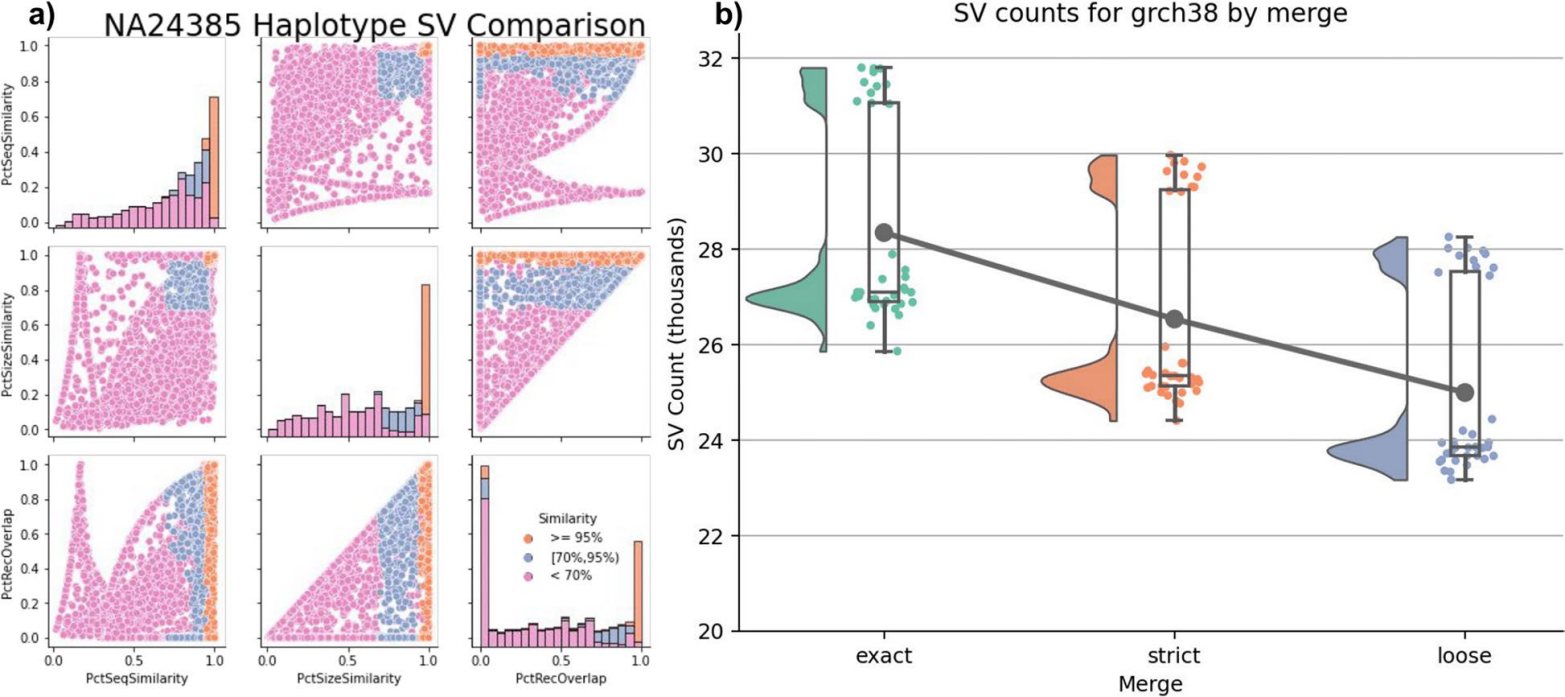
Intra-sample merging. **a** Distributions of similarity metrics of SVs between NA24385 haplotypes. Colors are thresholds for sequence and size similarity. **b** Effect of stringency on intra-sample merging SV counts for GRCh38. The trend line is the average number of SVs per merge. Separation of samples is attributable to ancestry

The simplest case of SV merging would be to combine SVs across haplotypes within a sample to create a diploid call set. At most, we expect a single match between haplotypes at homozygous alleles. For NA24385 on hg19, 5478 SVs from each haplotype have identical sequence and position and therefore comprise homozygous alleles. The remaining 20,719 SVs were compared using the Truvari *bench* to identify the similarity of SVs between haplotypes (Fig. 2a). This showed 1576 SV pairs having at least 95% sequence and size similarity and 1195 between 70 and 95% similarity, all of which are candidates for merging. Interestingly, 402 SV pairs have ≤ 5% reciprocal overlap but ≥ 70% sequence and size similarity. These pairs may indicate alignment ambiguities across repetitive regions (e.g., left shift vs. right shift).

We next investigated the effects of matching stringency on SV merging by creating three different merges: (i) exact method—the most stringent approach, combines SVs if their breakpoints, size, and sequence are identical; (ii) strict method—variants within 500 bp and over 95% sequence and size similarity are merged; (iii) loose method—variants within 1000 bp and over 70% similarity are merged. We used Truvari *bench* to compare the three NA24385 intra-sample merges to GIAB Tier1 SVs (Additional file 8: Table S1). If merging stringency played no role in the final results, we expect to observe no changes in the amount of variation shared between the GIAB benchmark and the diploid call set.

We observed 93.7% recall for the exact and strict merges and 93.6% recall for loose. To measure the effect the merges have on the resulting SVs, we count the ratio of how many true-positive (TP) GIAB SVs are lost and how many potentially redundant calls are removed from the strict and loose merges compared to the exact merge. We found a ratio of 1:790 for strict and 1:141 for loose. Only 4.1% of false negatives (FN) and 2.8% of false positives (FP) from strict have no complementary calls within 1000 bp. Therefore, with extremely permissive thresholds, strict could have up to ~ 96% recall and ~ 97% precision. The remaining 391 FNs (238 INS; 153 DEL) are partially explained by 37% lacking aligned coverage from the assemblies as well as being enriched for SVs ≥ 5000 bp (chi-square *P* < 1E−5).

As merging becomes more permissive, SVs are more likely to find a match between haplotypes, thus lowering the overall SV count (Fig. 2b). When looking across all 36 samples and all references, exact produces an average of 27,187 SVs per sample, whereas the strict and loose merging lower the average SV count by 1520 and 2851, respectively. Additionally, merging impacts the variant heterozygous vs. homozygous (het/hom) ratio due to Truvari consolidating the genotypes of heterozygous calls collapsing into a single homozygous variant (see methods) (Fig. S1). The average and standard deviation of het/hom ratios across samples and references are 4.9 ± 0.9 for exact merges, 3.2 ± 0.7 for strict, and 2.3 ± 0.5 for loose.

These patterns of merging’s effects on QC metrics appear on each reference and by SV types, though to differing degrees. When averaging the results across thresholds, for GRCh38, we see more SVs per sample (26.6K) than chm13 (24.4K). However, GRCh38 has an imbalance of SV type frequency, with more insertions (16.5K) than deletions (10.1K), whereas chm13 is almost balanced (11.9K DEL, 12.4K INS). The most drastic change in SV counts due to merging comes from GRCh38 insertions where loose merging results in a 15.9% decrease in SV count compared to exact (Fig. S2). As previously reported [21], we observe a greater number of SVs from individuals of African ancestry with an average of ~ 30.8K SVs compared to ~ 25.6K SVs from all other individuals (Fig. S3).

This analysis shows how even high-quality pipelines can produce multiple SV representations of the same allele. Furthermore, the changes in SV counts and het/hom ratios from increasingly permissive matching thresholds highlight the importance of careful SV comparison. The 95% sequence and size similarity thresholds from Strict merge have a well-balanced removal of redundant alleles and preservation of unique SVs across individual samples for this call set. Thus, we chose these thresholds for Truvari *collapse* to produce the final per-sample VCFs.

### SV merging’s impact across multiple samples

Next, we investigated how merging approaches perform across multiple samples to demonstrate their impact on the results. From the individual VCFs produced in the previous step, we created a project-level VCF (pVCF) across all samples for each reference using five SV merging tools: BCFtools, Truvari, Jasmine, Naive 50% reciprocal overlap, SURVIVOR. These tools use a variety of methods for SV comparison and represent a broad selection of the currently available SV merging approaches (Additional file 9: Table S2).

The relationship between decreasing matching stringency and decreasing SV count was established above. Here, BCFtools is the exact matching method and serves as an upper limit to which we compare the other tools since it retains all redundant variants and therefore holds the maximum possible number of SVs. BCFtools produces 347,158 SVs for GRCh38 (80,322 DEL; 266,836 INS) and 329,937 SVs for chm13 (121,038 DEL; 208,899 INS). The lower-limit average allele frequency (AF) from BCFtools is 0.05. Using Truvari *anno repmask* and *anno numneigh*, we observe the highest number of SVs per locus—and thus most likely in need of merging—is annotated as low complexity (average 8.5 SVs/locus) and simple repeats (6.7) (Fig. S4).

Relative to BCFtools, the merges have an average reduction in SV count of Truvari 41%, Jasmine 59.8%, Naive 65.2%, and SURVIVOR 77%. The largest difference in SV count reduction is between Truvari, which produces an average of 199,751 SVs, and SURVIVOR with 77,761. Broken down by SV type, this is a difference of ~ 38.5K DEL and ~ 83.5K INS. Additionally, the average AF observed in pVCFs is Truvari 0.08, Jasmine 0.12, Naive 0.13, and SURVIVOR 0.17. Therefore, choices in merging tools can cause an approximately 1.7× to 3.6× fold increase in AF. For details on SV count, average AF, and size distributions, see Additional file 10: Table S3, Fig. S5, and Fig. S6.

**Fig. 3:**
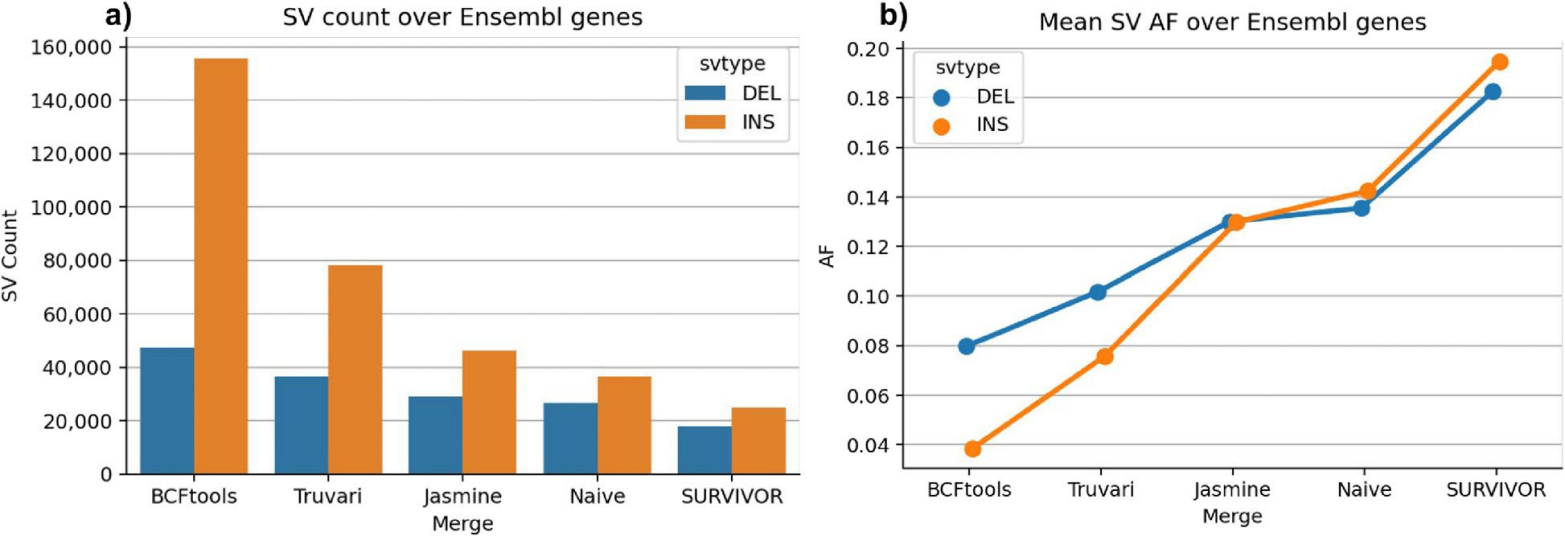
Merging strategies’ impact on pVCF number of SVs and their allele frequency over Ensembl genes. **a** Count of deletions and insertions produced by each merging strategy. **b** Average allele frequency of SVs as merging leniency increases

These patterns of SV count reduction and increased AF are not only present genome wide, but also within genes. To highlight this, we used Truvari *anno bpovl* to identify SVs which intersect genes from Ensembl release-105 [22] on GRCh38. A total of 155,722 insertions and 47,328 deletions from the BCFtools merge were found to have any overlap with genes. Figure 3 shows that Truvari produces more variants at a lower average AF compared to the other tools which attempt to remove redundant alleles.

### Benchmarking pVCFs

GIAB recently published an expanded benchmark of challenging, medically relevant gene regions (CMRG) [23]. This includes 273 genes on GRCh38 which were resolved for SVs in NA24385. In total, there are 216 SVs from NA24385 intersecting CMRG. Our SV calling pipeline identifies 2363 SVs in CMRG regions across all individuals. Using Truvari *bench*, we assess how well merging tools are preserving variants by comparing non-reference-homozygous NA24385 sites in the pVCFs against CMRG. Because BCFtools only merges identical alleles and makes no attempt to remove redundancy, it has the highest possible recall with 201 TPs. However, of the tools that remove redundant variant representations, we again see that Truvari’s pVCF is best at preserving variants with one TP missing whereas the remaining tools over-merge and lose between 5 and 41 TPs (Table 1). The manual analysis found that Truvari’s single lost TP was inside the RNF213 gene at chr17:80,274,587 where two heterozygous insertions of length 538 bp and 580 bp with 96.2% sequence similarity were merged.

**Table 1:** Merges’ pVCF performance on GIAB CMRG SV benchmark of NA24385 for GRCh38. Hardy-Weinberg equilibrium (HWE) and excess heterozygosity (ExcHet) scores less than 0.05 were counted for true positives across all 36 samples’ genotypes

Merge	TP	FP	FN	Precision	Recall	f1	Call cnt	gt_concordance	HWE < 0.05	ExcHet < 0.05
**BCFtools**	201	5	15	0.976	0.931	0.953	206	0.950	39	1
**Truvari**	200	5	16	0.976	0.926	0.950	205	0.950	17	4
**Jasmine**	196	10	20	0.951	0.907	0.929	206	0.949	17	4
**Naive**	171	22	45	0.886	0.792	0.836	193	0.947	26	18
**SURVIVOR**	160	28	56	0.851	0.741	0.792	188	0.956	24	20

Two metrics for evaluating the genotyping quality of variants are Hardy-Weinberg equilibrium (HWE) and excess heterozygosity (ExcHet) scores. Excluding variants with lower values of these scores is a common QC step in association studies [24]. Using NA24385 true positives from each merge, we calculated the HWE and ExcHet across all 36 samples’ genotypes with the idea that fewer calls with lower scores (< 0.05) indicate a higher-quality merge (Table 1). The smallest proportions of TPs with low HWE are from Truvari and Jasmine results at 8.5% and 8.7%, respectively. The largest proportion is from the under-merged BCFtools results at 19.4% of all TPs. For ExcHet, we find up to 2% of BCFTools, Truvari, and Jasmine variants having low scores compared to 10.5% of Naive and 12.5% of SURVIVOR TPs.

### Assessing the performance of merging tools

Beyond quantifying the differences of each merge, we need to assess how well they preserve measurably distinct alleles. The goal of SV merging is to identify redundant representations of alleles and consolidate their genotypes. Over-merging occurs when unique SV representations are falsely identified as being redundant. Ideally, a correct merge would retain all unique alleles while consolidating only truly redundant alleles.

**Fig. 4:**
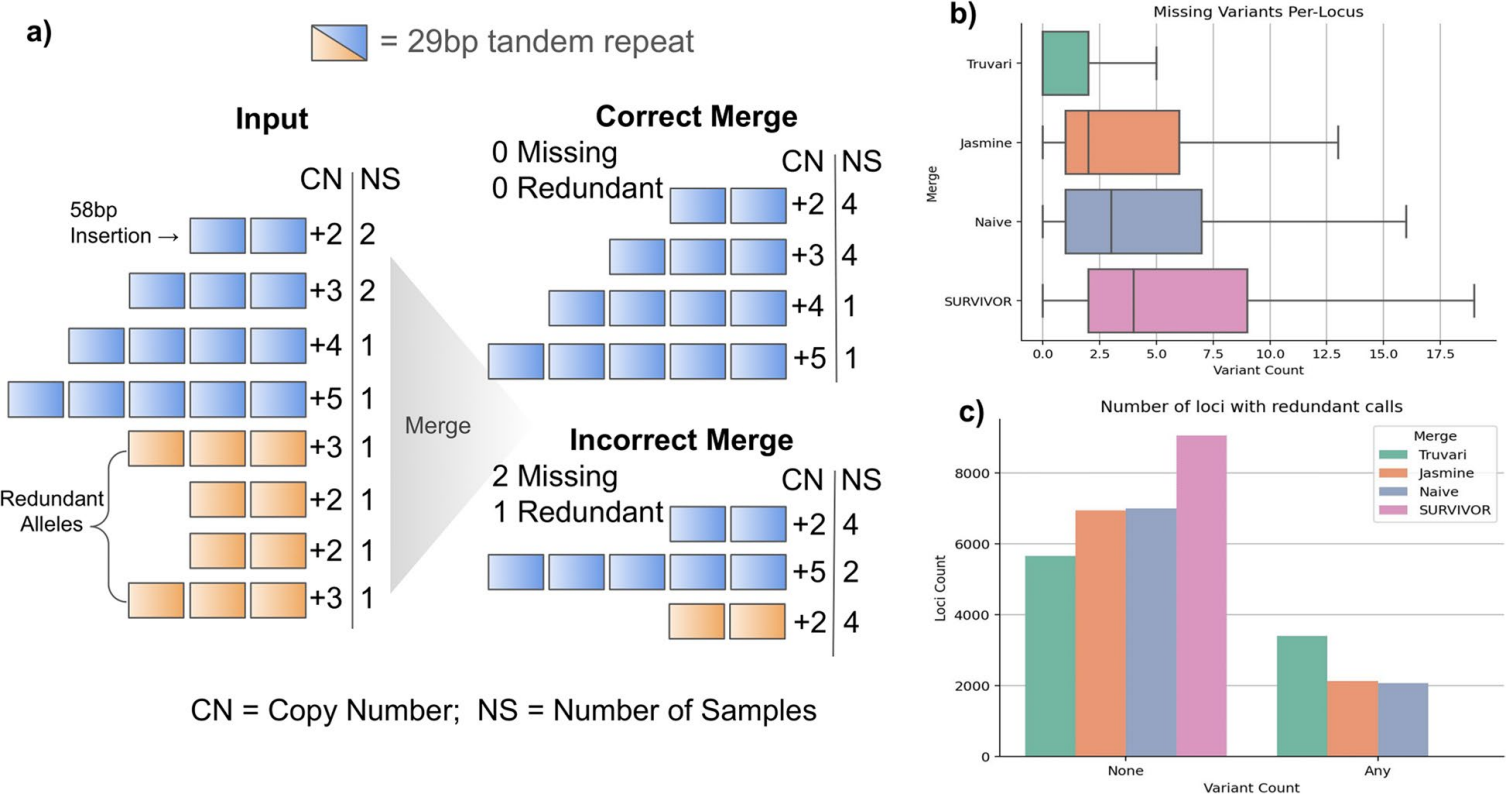
Investigation of tandem repeats to assess merging strategies’ performance. **a** Illustration of a locus where eight insertion alleles (Input) have between + 2 and + 5 copies of a 29-bp repeat across 10 samples. Four of the alleles are annotated as redundant representations (blue) since they have a counterpart with an equal number of copies (orange). A correct merge would preserve each of the unique alleles and remove all redundant alleles, leaving 0 missing and 0 redundant SVs in the locus. An incorrect merge removes two unique insertions (+ 3, + 4) and leaves 1 redundant insertion. **b** Boxplot of the number of missing variants per locus for each merging strategy. **c** Barplot of the number of loci with none or any redundant alleles post-merging

One case where we expect an enrichment of redundant SV representations is in tandem repeat regions due to alignment ambiguities. Furthermore, we can classify all variants in a tandem repeat locus as representing unique or redundant expansions/contractions of the reference by running TandemRepeatFinder (Fig. 4a, see the “Methods” section). Since BCFtools performs exact matching and only identical alleles are consolidated, it preserves every input allele but fails to consolidate genotypes between redundant representations. Consequently, we can use BCFtools’ result as a baseline to which we compare in order to assess how many unique alleles are missing (over-merging) and how many redundant alleles remain (under-merging) in the SV merging tools’ results (see the “Methods” section).

We identified 20,207 tandem repeat loci with SVs. Of these tandem repeat loci, 9056 (44%) have a different number of SVs reported from at least one merging tool. Thus, this subset of highly problematic regions was analyzed to assess the amount of missing alleles (Fig. 4b) and redundant alleles (Fig. 4c) in the merge tools’ pVCFs. Truvari had over-merging in 47.4% of loci (average of 1.8 missing alleles per locus). Jasmine and Naive had over-merging in 76.5% (4.4 alleles per locus) and 79.6% (5.4) of loci, respectively. SURVIVOR, the most permissive SV merging tool, exhibited over-merging in 99.3% of loci, which averaged to 7.1 missing alleles per locus. For the loci with redundant alleles remaining Truvari, produces 3398 loci (37.5%) having at least 1 redundant allele compared to BCFtools, Jasmine 2118 (23.3%), Naive 2058 (22.7%), and SURVIVOR produces 13 loci (0.1%) with redundant alleles.

This analysis shows that using orthogonal information, we can objectively demonstrate that of the tools which attempt to identify and consolidate redundant allele representations, Truvari is performing best while other tools are over-merging more frequently, which in turn inflates AF and loses unique alleles.

### Computational performance and generalizability

To compare the computational performance of the merging tools, we collected SV calls for 33 samples from two short-read SV discovery programs Manta [15] and BioGraph [14] (see the “Methods” section). Using a single core and 4 gigabytes of ram for each analysis, the fastest tools on average were BCFtools and SURVIVOR which took approximately 19 s while the slowest tool was Jasmine which took 15 min. Truvari only took 5 min (Additional file 11: Table S4). Figure S7 shows the SV counts in the pVCFs produced by each merging tool over the two short-read discovery tools.

The merging results from short-read discovered SVs (Fig. S7) show a similar trendline to the long-read discovered SVs in that as merging becomes more permissive, fewer variants are produced in the pVCF. This suggests all the merging tools are generalizable to SVs produced by multiple sequencing technologies. However, the differences between the merging tools’ results on BioGraph and Manta are less drastic as there are fewer insertions called on short reads.

## Discussion

A goal of genome analysis is to precisely resolve all SVs at the nucleotide level in order to improve the understanding of the mechanisms of their origin and their biological impact. Here, we describe Truvari, a toolkit that enables merging, benchmarking, and annotation of SVs. We showed Truvari’s versatile applications to SV analysis and how it significantly improves the ability of researchers to accurately compare structural variants. We demonstrated this across an SV call set for 36 haplotype-resolved long-read assemblies [16, 17] by starting with the simplest case of SV merging and identifying identical alleles between haplotypes before progressively allowing more lenient matching with more permissive SV comparison thresholds. We observed the expected pattern of more lenient thresholds predicting more matching SVs between haplotypes. As the problem of SV merging becomes more complex when merging between samples, we showed how Truvari’s approach outperforms other tools at preserving distinct alleles genome wide, within genes, and in especially problematic tandem repeats. Throughout the project, we measured the performance of the SV calls with comparisons to GIAB SV benchmarks using Truvari [6, 23].

This research focused on SVs generated by long-read assemblies in order to demonstrate the complexity of SV comparison and merging even given a “best case scenario” of input SV calls. However, Truvari is not restricted to input SVs produced from phased assemblies. Truvari’s flexibility allows it to be used on any VCF with SVs, even those generated by short reads as demonstrated in the section on computational performance. It is important to note that Truvari is currently most useful for “resolved” SVs (i.e., DEL, INS, INV, and DUP). What we have not addressed in this manuscript are the challenges of multi-technology or unharmonized pipeline-based SV comparison. The similarity of SVs is highly dependent on the study design itself as call sets can report SVs with imprecise breakpoints or lacking of sequence resolution (e.g., optical mapping, HiC sequencing). There are reasons for optimism since across sequencing technologies there is continued development of SV detection methods that report the sequence resolved, breakpoint exact information needed to fully differentiate SVs [14, 15, 25]. Further work is needed to comprehensively solve the challenges introduced by genomic loci harboring complex genomic rearrangements [26] or pipelines producing highly disparate SV representations. But in the work described here, investigating the most common cases encountered, Truvari is demonstrated to accurately resolve SV comparisons which other methods mishandle.

We assessed the impact of merging tools on a single sample up to the population level. In one experiment on the latter, we measured the genotype quality of pVCFs produced by the merging tools with Hardy-Weinberg Equilibrium (HWE) and excess of heterozygosity (ExcHet) scores. The threshold (0.05) used for calling a variant as “ExcHet” is likely slightly over-conservative in this experiment as the general effect of population structure in this sample is to decrease rather than increase heterozygosity compared to Hardy-Weinberg expectations. Meanwhile, the two-tailed HWE is likely slightly, but not strongly, under-conservative. In either case, for a sample of this size (*n* = 36), only very strong deviations achieve the threshold of statistical significance and are much more likely to be driven by technical as opposed to demographic factors.

Given the limited set of SVs that are fully resolved, it is unknown when alleles with high sequence similarity should remain unmatched. This is highlighted in our experiments which assessed the performance of the merging tools. We assumed in this work that small sequence differences in alleles were due to sequencing errors, but some of these changes may represent biologically relevant differentiation. The tandem repeat performance assessment identified Truvari as having the highest count of loci with redundant alleles remaining after the merge. However, these “redundant” alleles may be explained by point mutations in a copy of the tandem repeat such that TRF can still identify the repeat, but the sequence is different enough to have biological consequences such as inhibiting the tandem repeat’s slipped-strand mispairing mechanisms [27]. If this is the case, these “redundant” alleles should not be considered the same because an allele without accumulated point mutations may be more susceptible to further contraction/expansion of the tandem repeat than an allele with mutations. We are therefore investigating how dynamic thresholding can further improve Truvari’s SV comparison accuracy.

The overall importance of correct SV comparison is clear. One of the most remarkable results from Truvari is the shift in allele frequencies across the spectrum of merging tools. Figure 3 showed that other methods’ over-merging has a large impact on allele frequency, particularly for insertions. These differences have drastic implications on the interpretation of SVs across a population since sequence differences between individuals are getting lost. Previous publications suggest a potential over-merge, but further investigations are needed to address the fidelity of data emerging from the rising number of studies investigating insertions, particularly those using long reads [28–32].

Finally, data from Truvari informs the important question as to the overall number of SVs that one might assume to be present in the genome of healthy humans (Fig. 3a, Fig. S5). Using these phased assembly-based SV call sets, we conclude that the number of SVs per human might be higher than previously suggested, which again highlights the importance of this class of genomic variation.

## Conclusions

The choices made when performing SV comparisons have important impacts on the results. When SV comparison is too lenient, over-merging occurs, distinct alleles are lost, and metrics such as allele frequency are inflated. This research shows how Truvari’s method of leveraging multiple SV similarity metrics enables refined handling of SV comparison and a better approach to multi-sample SV analysis.

## Methods

### Truvari SV comparison

Truvari’s core functionality (Fig. 1) involves building a matrix of pairs of SVs and ordering the pairs to determine how each should be handled. To start, VCFs are consolidated using a “zipper.” This procedure opens sorted VCFs using pysam (a wrapper around htslib). The set of VCFs is then treated as a single stack where the ascending alphanumeric sorting of each chromosome and integer position is yielded by a generator. This zipped stack of variants is “chunked,” and all variants within a *chunksize* are grouped. The chunker is also responsible for variant filtering on properties such as size restrictions, reference location, or VCF FILTER as specified. Chunks are created between sets of variants where the maximum end position plus chunk size is greater than the start position of the next variant yielded from the zipper. The zipping and chunking infrastructure is reused for *bench* and *collapse*. Additional filtering parameters such as only comparing passing variants or those genotyped as being present (non-reference-homozygous) in a sample being analyzed are available and prevent calls from being used downstream.

The next step for the *bench* procedure is to build an NxM matrix of the baseline and comparison calls within a chunk of variants. If dimensions N or M are 0, all variants within the chunk are annotated as false negatives (FN) or false positives (FP), respectively. Each pair is then measured for similarity across multiple metrics to build a putative match.

Variants have the properties of start position (*S*), end position (*E*), length (*L*), and allele sequence (*A*). Deletion’s *L*(*A*) = *E* − *S* whereas insertions have no span over the reference and length is simply *L*(*A*). Formal definitions of each metric follow:

**Reference distance:** Variant’s positions are within the specified *refdist*:

$$\max \left({S}_1- refdist,{S}_2\right)<\mathit{\min}\left({E}_1+ refdist,{E}_2\right)$$

**Reciprocal overlap:** Percent of overlapping bases over the maximum variant span

$${\displaystyle \begin{array}{l}{O}_s=\max \left({S}_1,{S}_2\right)\\ {}{O}_e=\min \left({E}_1,{E}_2\right)\\ {} rec\_ ovl=\left\{\begin{array}{c}\frac{\left({o}_e-{o}_s\right)}{\max \left({E}_1,{S}_1,{E}_2,{S}_2\right)}\\ {}0\end{array}\right.\kern0.5em \begin{array}{c} if\ {o}_s<{o}_e\\ {} if\ {o}_s>{o}_e\end{array}\end{array}}$$

**Size similarity:** Minimum variant length over the maximum variant length:

$$\frac{\min\ \left(L\left({A}_1\right),L\left({A}_2\right)\right)}{\max\ \left(L\left({A}_1\right),L\left({A}_2\right)\right)}$$

**Sequence similarity:** Haplotype sequence similarity calculated with edlib [33]:

$${\displaystyle \begin{array}{l} start=\min \left({S}_1,{S}_2\ \right)\\ {} end=\max \left({E}_1,{E}_2\right)\\ {}\begin{array}{c}{H}_1= ref\left[ start:{S}_1\right]+{A}_1+ ref\left[{E}_1: end\right]\\ {}\begin{array}{l}{H}_2= ref\left[ start:{S}_2\right]+{A}_2+ ref\left[{E}_2: end\right]\\ {}\begin{array}{l} edit\_ distance= edlib\_ align\left({H}_1,{H}_2\ \right)\\ {}\begin{array}{l} totlen=L\left({H}_1\right)+L\left({H}_2\right)\\ {} seqsim=1-\left( edit\_ distance/ totlen\right)\end{array}\end{array}\end{array}\end{array}\end{array}}$$

The reciprocal overlap of sequence-resolved insertions, which have no physical span over the reference, is measured after the event’s boundaries are expanded by half the SV’s length upstream and downstream. To compute sequence similarity, the span of reference sequence between the two SV’s upstream-most start and downstream-most end is fetched and the sequence change of the SV is incorporated to create the shared sequence context of the calls. The two sequences are then aligned and similarity reported. The reciprocal overlap, size similarity, and sequence similarity metrics are averaged to create a TruScore for ranking of putative matches. Each putative match is assumed to be valid until a comparison fails the thresholds/flags provided by the user. Additionally, the distance between the start and end breakpoints of the pair of calls is recorded for annotation purposes.

Once the matrix of putative matches is filled, it can be used to identify the best matches between the baseline and comparison calls. By default, only the single best match is searched for by raveling the 2D matrix into a 1D array and sorting the putative matches by their TruScore. Each match’s calls are checked to ensure they have not been used in a previous match. If neither have the putative match with its state as determined by the thresholds is passed along to the output. If either call has been used previously, the match’s state is set to false and the unused baseline/comparison call in the pair is output as FP/FN, respectively.

In some cases, a user may wish to allow variants to participate in more than one match. For example, one may expect multiple representations of an SV from a caller where there is only one inside the baseline variants. In this case, parsing the match matrix involves sorting each row and column independently by the TruScore such that the highest scoring match for each baseline and comparison call is reported.

For Truvari *collapse*, the same procedure to build matches is employed; however, instead of a matrix of baseline/comparison, we have an NxN matrix of all calls within the chunk. Additionally, two more parameters are checked when building the match. If the user specified *--hap*, incompatible intra-sample genotypes are unable to be a valid match, e.g., homozygous alternate calls in the same individual are not matched. Without this parameter, genotypes are consolidated such that, e.g., two heterozygous variants become a single homozygous variant. The second parameter unique to Truvari *collapse* is *--chain*, which allows more flexibility around the *--refdist*. Chaining allows transitive matching such that two variants that do not directly match but have a shared intermediate match are considered matching. After the matches have been built, each set of matching variants is sorted to determine which variant is kept in the output as the representative variant while the remaining are written to an extra VCF of collapsed variants. The options of which variant to keep from a set are as follows: first, the most upstream variant; maxqual, the variant with the highest QUAL score; and common, the variant with the highest minor allele count.

### Reference genomes

Human genome 19 (hg19), GRCh38, and telomere-to-telomere consortium chm13 v1.0 references were downloaded [18–20]. Alternate contigs were removed, and variant calling was performed against only autosomes and the sex chromosomes X/Y.

### SV calling

Previously published long-read, haplotype-resolved assemblies [16, 17] were mapped with minimap2 [34] version 2.17 and variants called with paftools, which is part of the minimap package. Minimap2 parameters used were “*-cx asm5 -t8 -k20 --secondary=no --cs ${ref} ${fasta}*” and paftools parameters “*-L10000*.” Three individuals (HG00733, NA12878, NA24385) had assemblies created by both projects. In those cases, we chose to keep the assemblies generated by Garg et. al. as an attempt to increase the heterogeneity of variants which would further test merging.

### Intra-sample haplotype merging

VCFs produced per haplotype for each individual were merged using BCFtools v1.13 [35]. A custom script consolidated genotypes to create a single SAMPLE column per VCF. Truvari *collapse* v3.1 was run with *--hap* to prevent incompatible genotyped calls from being merged to produce the “strict” intra-sample merge. Truvari *collapse* v3.1 parameters to produce the “loose” merge “*--hap --pctsim 0.70 --pctsize 0.70 --refdist 1000*.” VCFs were converted to pandas DataFrames using Truvari *vcf2df* for analyses which can be recreated using the project’s GitHub.

### RepeatMasker classifications

Truvari *anno repmask* is a wrapper around RepeatMasker [36] that adds the annotation information into a VCF. For deletions, the REF sequence is run through RepeatMasker whereas for INS, the ALT sequence is used. For this study, a minimum RepeatMasker score of 250 was required to accept a reported annotation.

### Number of neighbors

Truvari *anno numneigh* annotates entries in a VCF with how many other entries are within a specified distance as well as assigning an identifier for all variants within the same genomic region (i.e., neighborhood) as defined by the specified distance.

### GIAB benchmarking

Comparisons to Genome in a Bottle consortium’s SVs v0.6 were performed against hg19 [6] over the Tier1 regions. Comparisons to GIAB’s challenging, medically relevant genes (CMRG) SVs v1.0 were performed against GRCh38 over the resolved regions bed [37]. Truvari *bench* defaults were used. Hardy-Weinberg Equilibrium (HWE) and excess heterozygosity scores (ExcHet) were calculated using BCFtools +fill-tags.

### Inter-sample merging

Project-level VCFs were created using the per-sample VCFs generated by Truvari *collapse* with default parameters. BCFtools [35] version 1.13 had parameters “*-m none -0*.” Truvari *collapse* was run with *--chain* and default parameters. Jasmine [13] v1.14 was run with parameters “-*-output_genotypes --default_zero_genotype*.” SURVIVOR [11] v1.07 was run with parameters “*1000 1 1 0 1 50*.” Naive merging is performed by a custom script (available on the GitHub) that merges variants with 500 bp and with ≥ 50% reciprocal overlap. Since insertion calls have no physical span over the reference (i.e., they exist between two reference bases), the naive merging expands their boundaries to ± (SVLEN//2). Allele frequencies within pVCFs were calculated using BCFtools *+fill-tags*. For most analyses, Truvari *vcf2df* was run to turn pVCFs into pandas DataFrame. Jupyter notebooks detailing steps of the analysis on GitHub.

### Gene intersection

Truvari *anno bpovl* was run to intersect pVCF entries to Ensembl release-v105 [22] on GRCh38. This tool creates an interval tree for each range in the annotation file and checks variants’ intersection at the breakpoints as well as reporting if a variant is contained within or completely overlaps annotation file entries.

### Tandem repeat experiment

Truvari *anno trf* incorporates a wrapper around tandem-repeat finder (TRF) [38]. We ran Truvari *anno trf* to annotate all SVs on GRCh38 that intersected the SimpleRepeats track procured from UCSC Table Browser [31]. Each intersecting variant is used to alter the SimpleRepeat reference region to reconstruct the sample’s haplotype. TRF then detects the repeat sequence and copy number difference in an alternate allele relative to the reference (e.g., + 5 copies of a 50-bp repeat comprise a 250-bp insertion). The longest tandem repeat found inside the altered sequence that’s shared with the reference annotations is reported as well as the copy number difference of the variant compared to the reference track. SV calls are grouped into loci using Truvari *anno numneigh* where variants within 1000 bp are clustered. For each locus, variant calls with identical tandem repeat annotations (motif and copy number) are labeled as redundant. This procedure of annotating variants and generating loci groupings is repeated across each pVCF produced by the merging tools. The BCFtools merge result serves as the baseline since it holds the maximum number of variants possible. Variant calls remaining at each locus from the tools which attempt to remove redundant representations are then compared to the loci produced by BCFtools. Variants are classified as “redundant” if more than one variant is annotated with identical motif and copy number. Variants are classified as “missing” if no variants in a locus hold a tandem repeat annotation which was present in the original BCFtools merge. In order to emphasize the differences between tools, loci with identical results across all merging tools (i.e., differences in merging approaches had no affect) are excluded. Each merging tool’s result is assessed per locus and variant representations with unique tandem repeat annotations missing or redundant representations remaining in the post-merge result are tallied.

### Computational performance

Manta VCFs on grch38 were downloaded from https://aws.amazon.com/blogs/industries/dragen-reanalysis-of-the-1000-genomes-dataset-now-available-on-the-registry-of-open-data/. From that same source, the BAMs were downloaded and run through BioGraph v7.1 against grch38, chm13, and hg19. Project-level VCFs were created for each tool/reference combination using each SV merging tool. Tools were given a single core for processing and 8GB of ram. Wall times were collected using the unix `time` command.

## Supplementary Information

**Additional file 1: Figure S1.** Het/Hom Ratios of Per-Sample VCFs by SVTYPE. Each point is a sample. Point colors are the intra-sample merge strategy. Shapes are references. As matching thresholds become more lenient, more heterozygous alleles find a counterpart and become homozygous, thus lowering the het/hom ratio. We see the ratios of INS (y-axis) dropping more quickly than DEL (x-axis).

**Additional file 2: Figure S2.** SV counts across inter-sample merges by SVTypes for GRCh38 and chm13. As matching thresholds become more lenient, more heterozygous alleles find a counterpart and become homozygous, thus lowering the SV count. We see a steeper decrease in INS counts than DELs, particularly for GRCh38.

**Additional file 3: Figure S3.** SV counts per-sample across merge strategies and references. Colors are sample’s population code. Samples from individuals of African ancestry have more SVs.

**Additional file 4: Figure S4.** SVs per-locus by RepeatMasker class.

**Additional file 5: Figure S5.** SVCount (a) and Allele Frequency (b) for 5 SV merging tools (columns) across references (x-axis). We note very minor differences between hg19 and GRCh38.

**Additional file 6: Figure S6.** SVCount by size-bins (x-axis) for 5 SV merging tools (columns) across references (rows).

**Additional file 7: Figure S7.** Trendlines of SV merging tools’ results for inputs produced by long-reads (Assemblies) and short-reads (BioGraph, Manta) across references. Note that chm13 results were not generated for Manta. Additionally, SURVIVOR failed to merge the Manta results.

**Additional file 8: Table S1.** GIAB v0.6 Tier1 SVs performance of the three intra-sample merges on hg19. TP-base: number of GIAB SV calls re-identified; TP-call: number of SV in call-set matching with GIAB SV calls; Precision: TP call / call cnt; Recall: TP base / (base cnt); F1: 2 * ((recall * precision) / (recall + precision)); base cnt: total number of GIAB SVs; call cnt total number of call-set SVs.

**Additional file 9: Table S2.** Inter-Sample Merging tools’ versions and description.

**Additional file 10: Table S3.** Details of inter-sample merging’s effect on SV counts and allele frequencies.

**Additional file 11: Table S4.** Runtimes of SV merging approaches given a single core. The missing SURVIVOR grch38 manta walltime is due to a failure by the software to produce a result.

**Additional file 12: Table S5.** Sample metadata.

**Additional file 13: Table S6.** Paths to long-read assemblies.

**Additional file 14: Table S7.** Paths to per-sample VCFs produced by Truvari collapse.

**Additional file 15: Table S8.** Paths to per-sample pVCFs produced by Truvari collapse.

**Additional file 16: Table S9.** Paths to short-read BAM files.

**Additional file 17: **Review history.

## Data Availability

Truvari is available on https://github.com/ACEnglish/truvari [39] under an MIT License. Detailed documentation on all of Truvari’s tools can be found on GitHub’s wiki at https://github.com/ACEnglish/truvari/wiki. This paper used version 3.1 which is readily available through the tagged version of the GitHub and distributed through pypi. Additionally, the version of the software used in this publication is available through Zenodo [40]. Analysis methods used in this manuscript can be reproduced by code available on a separate GitHub repository [41]. The README has details for commands to create all the data for analysis. The “manuscript/” folder has post-processing scripts and jupyter notebooks to recreate figures and results in this paper. Metadata for samples used by this analysis can be found in Additional file 12: Table S5. Full download paths to the raw long-read assemblies used can be found in Additional files 13: Table S6. Download paths to the project’s final Truvari produced per-sample VCFs and pVCFs can be found in Additional files 14 and 15: Table S7 and Table S8. Download paths to the BAMs with shot-reads aligned to GRCh38 can be found in Additional files 16: Table S9.
