# Supplementary figures and images for: Truvari: refined structural variant comparison preserves allelic diversity
Source: Genome Biol. 2022 Dec 27;23:271. doi: 10.1186/s13059-022-02840-6 (PMC9793516; doi:10.1186/s13059-022-02840-6)

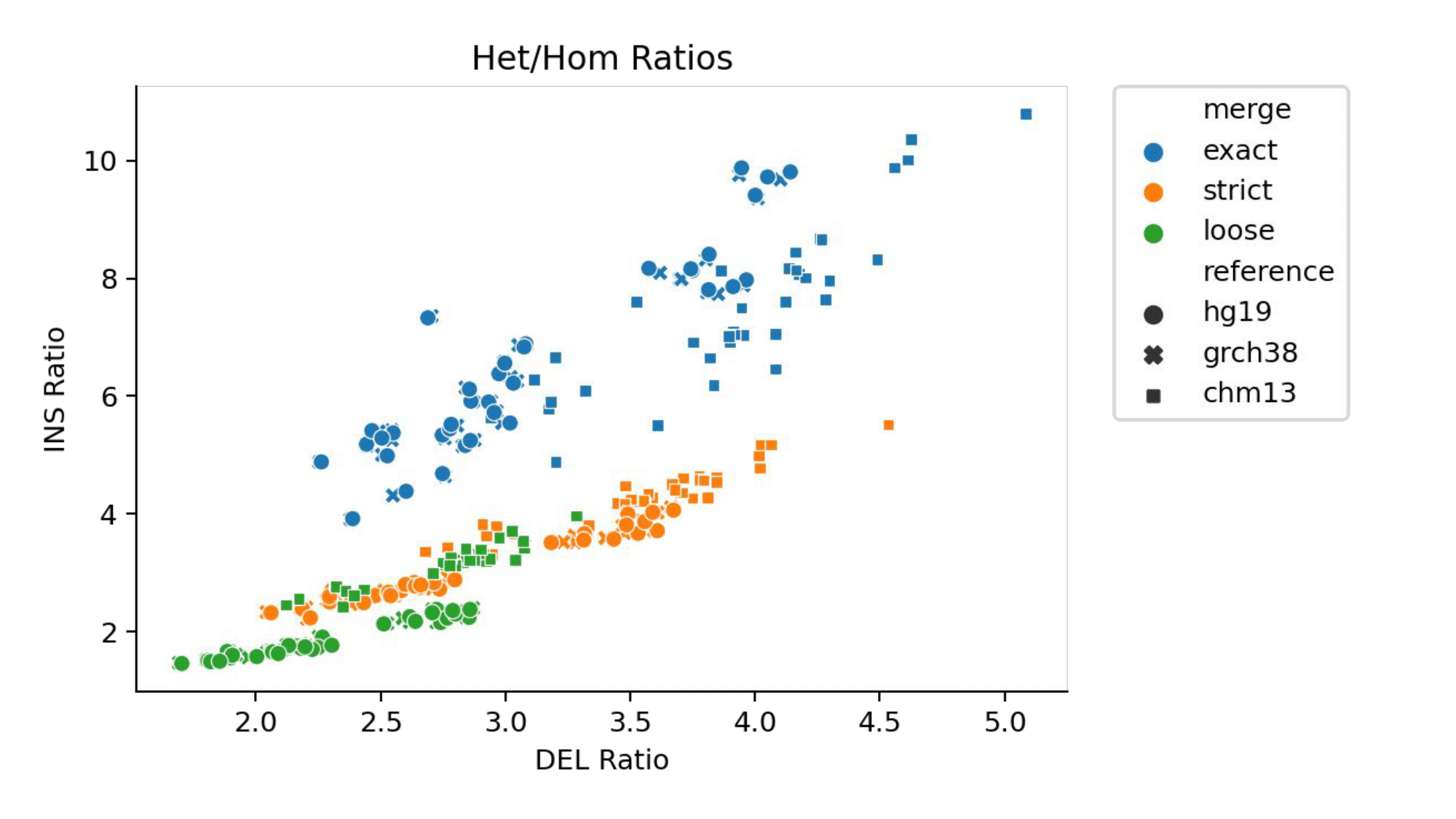

Supplement: Supplementary file 1 — Additional file 1: Figure S1. Het/Hom Ratios of Per-Sample VCFs by SVTYPE. Each point is a sample. Point colors are the intra-sample merge strategy. Shapes are references. As matching thresholds become more lenient, more heterozygous alleles find a counterpart and become homozygous, thus lowering the het/hom ratio. We see the ratios of INS (y-axis) dropping more quickly than DEL (x-axis). [file 13059_2022_2840_MOESM1_ESM.png]

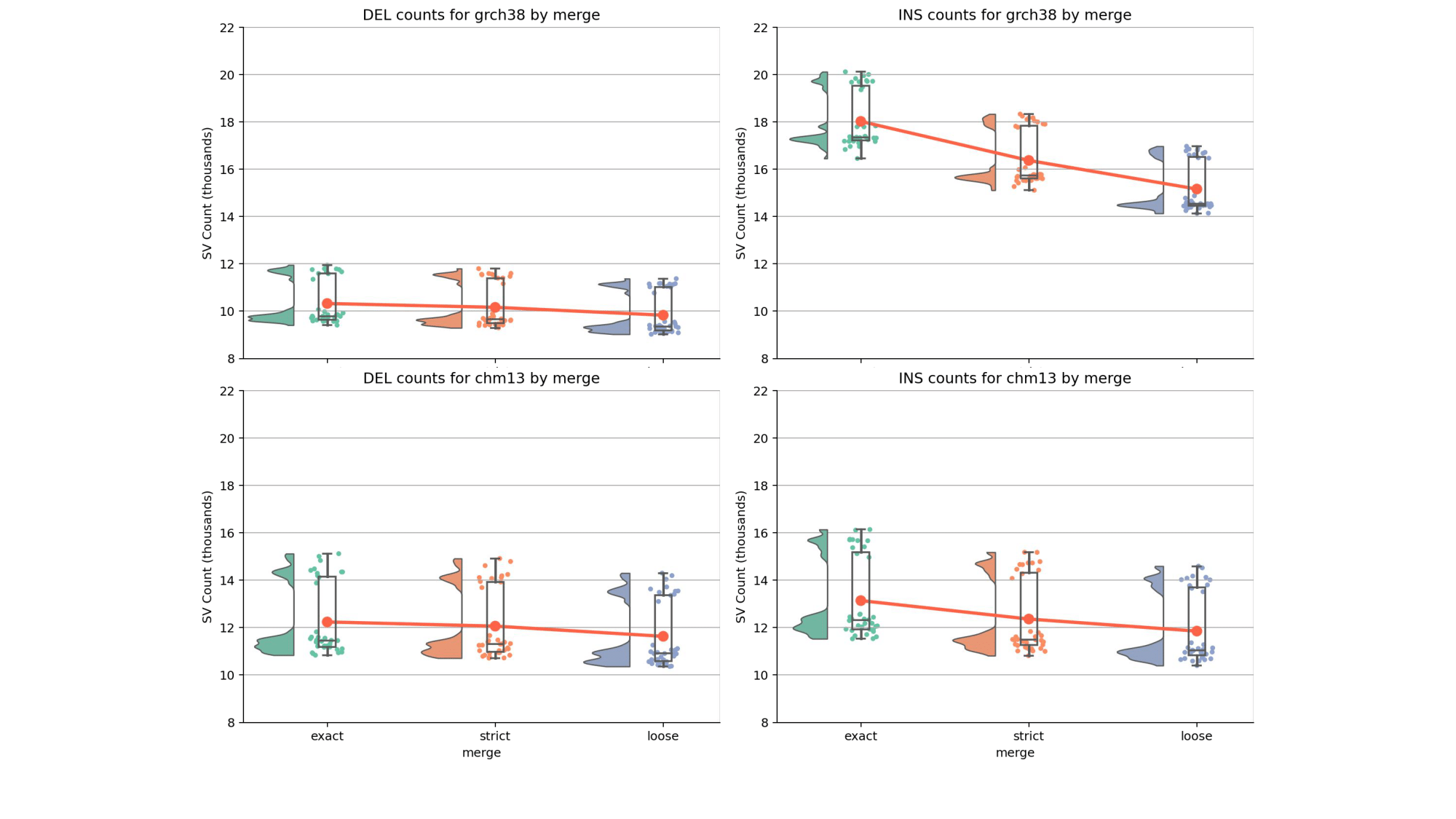

Supplement: Supplementary file 2 — Additional file 2: Figure S2. SV counts across inter-sample merges by SVTypes for GRCh38 and chm13. As matching thresholds become more lenient, more heterozygous alleles find a counterpart and become homozygous, thus lowering the SV count. We see a steeper decrease in INS counts than DELs, particularly for GRCh38. [file 13059_2022_2840_MOESM2_ESM.png]

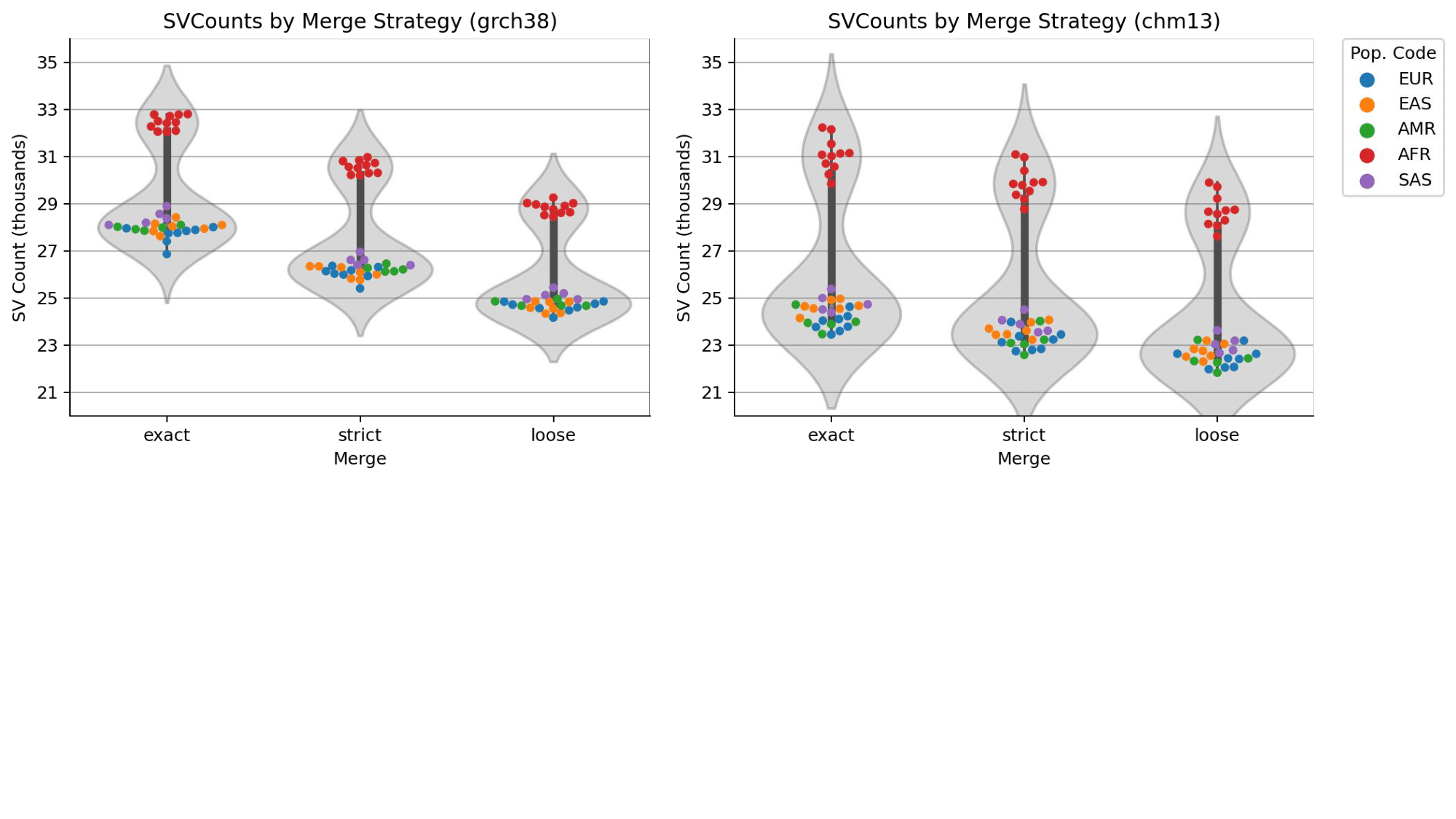

Supplement: Supplementary file 3 — Additional file 3: Figure S3. SV counts per-sample across merge strategies and references. Colors are sample’s population code. Samples from individuals of African ancestry have more SVs. [file 13059_2022_2840_MOESM3_ESM.png]

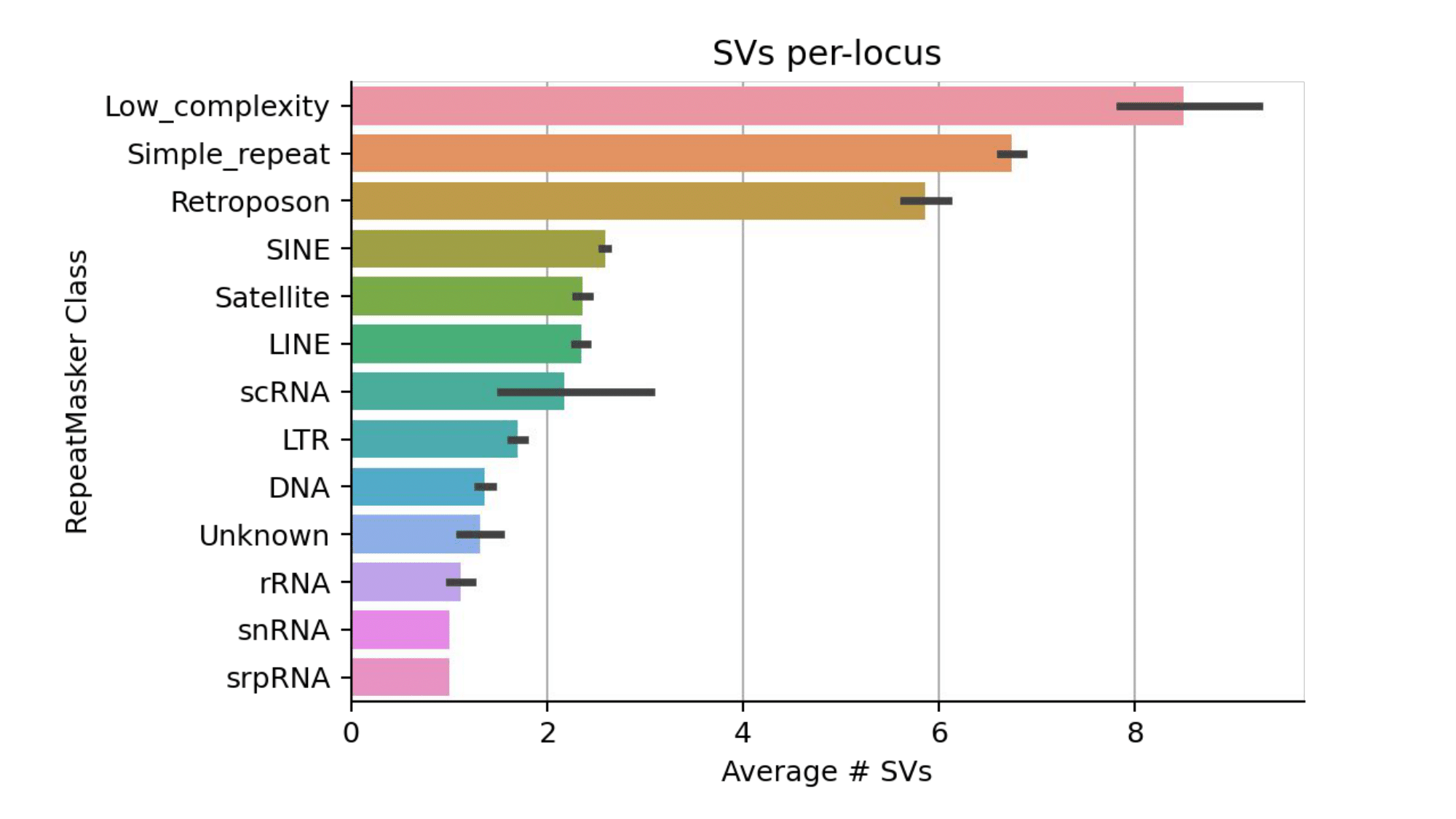

Supplement: Supplementary file 4 — Additional file 4: Figure S4. SVs per-locus by RepeatMasker class. [file 13059_2022_2840_MOESM4_ESM.png]

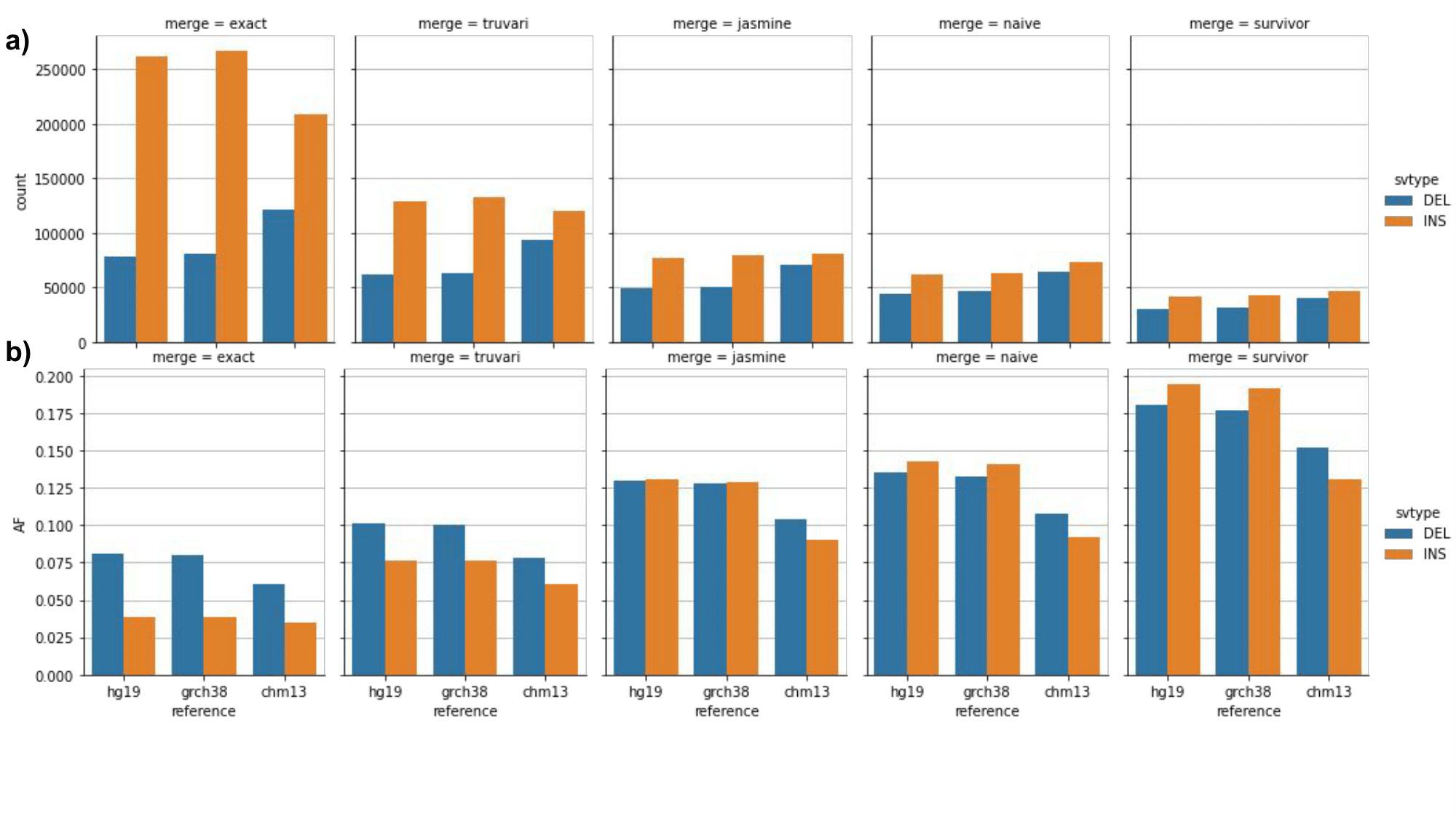

Supplement: Supplementary file 5 — Additional file 5: Figure S5. SVCount (a) and Allele Frequency (b) for 5 SV merging tools (columns) across references (x-axis). We note very minor differences between hg19 and GRCh38. [file 13059_2022_2840_MOESM5_ESM.png]

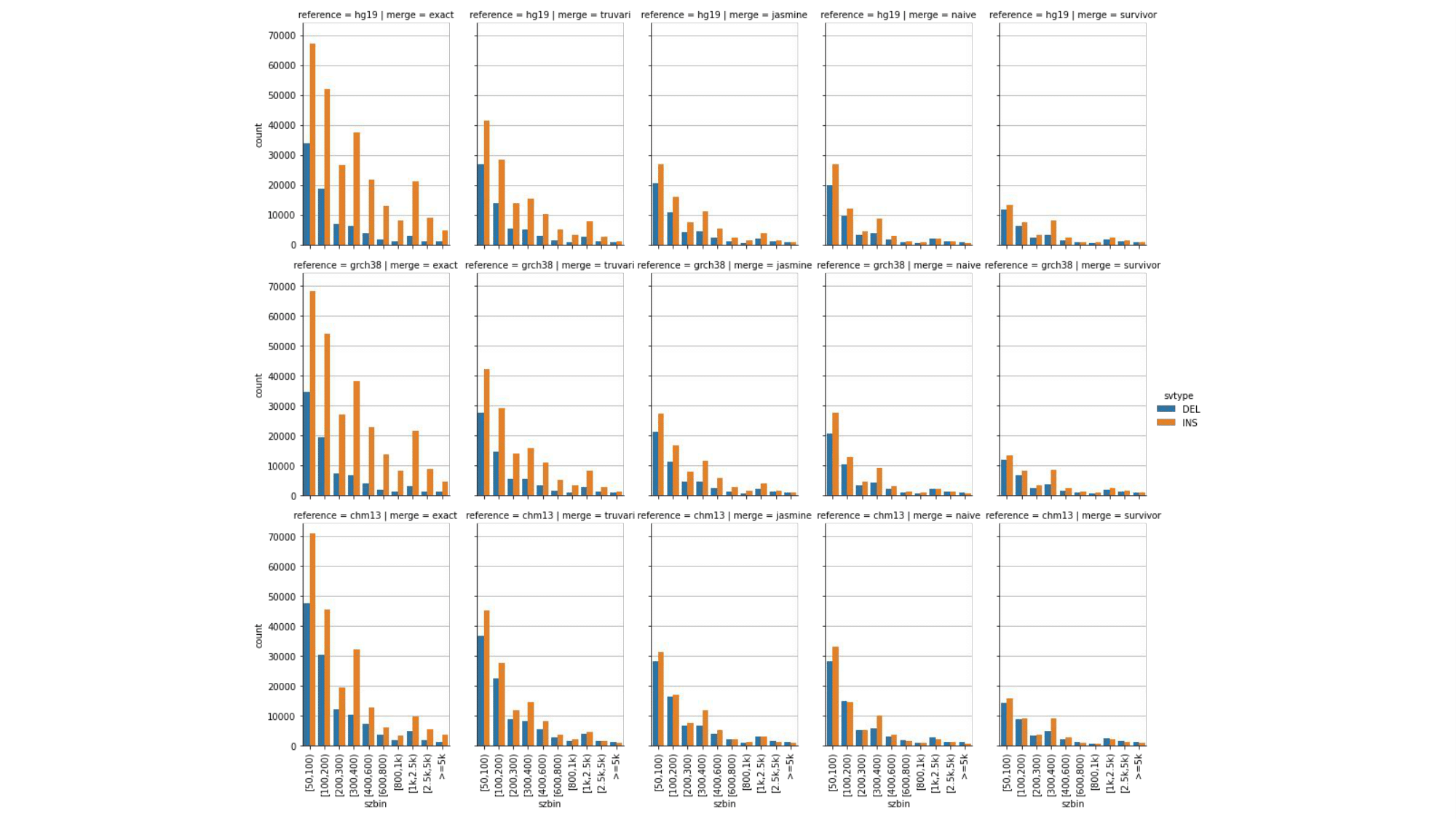

Supplement: Supplementary file 6 — Additional file 6: Figure S6. SVCount by size-bins (x-axis) for 5 SV merging tools (columns) across references (rows). [file 13059_2022_2840_MOESM6_ESM.png]

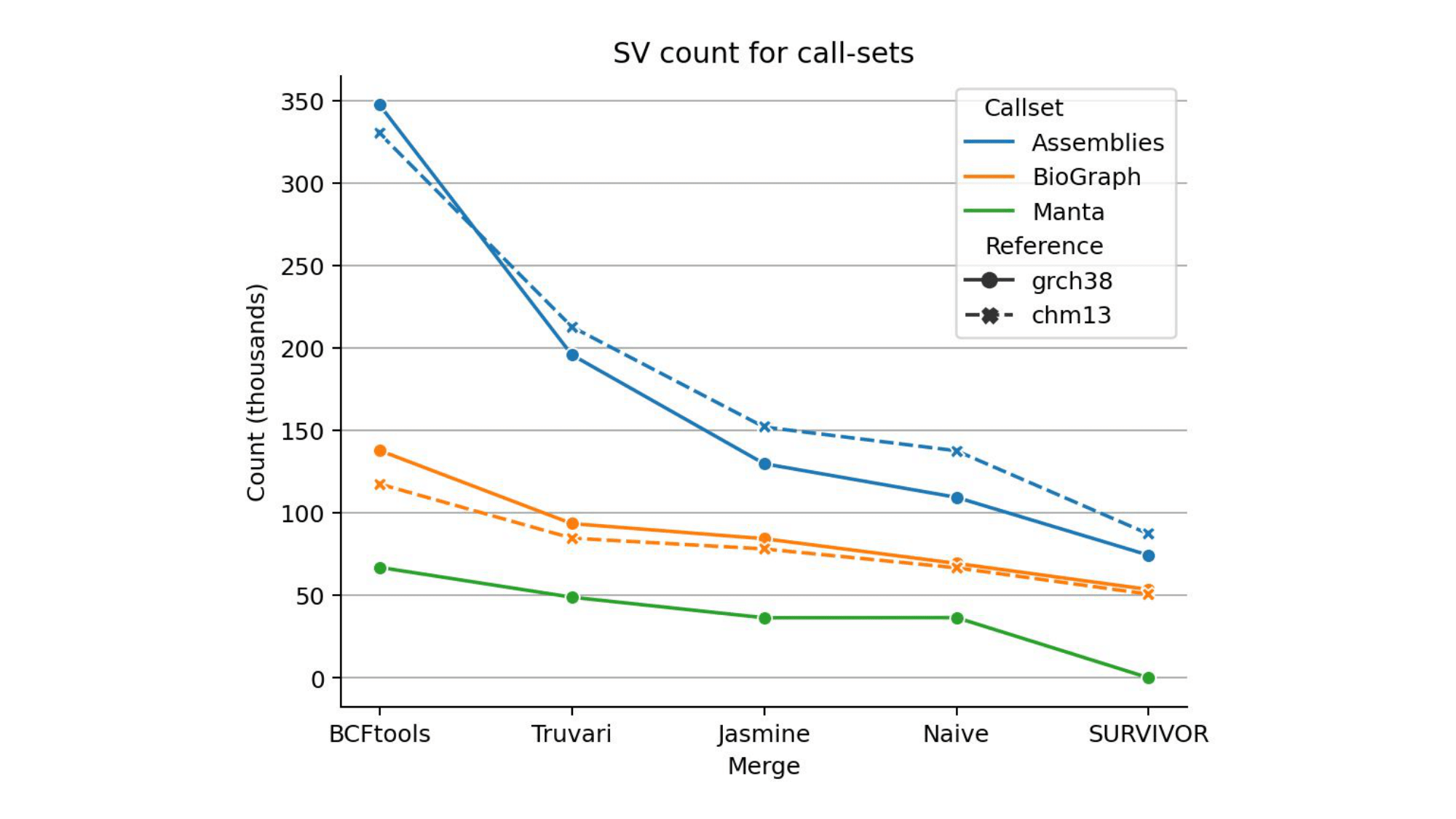

Supplement: Supplementary file 7 — Additional file 7: Figure S7. Trendlines of SV merging tools’ results for inputs produced by long-reads (Assemblies) and short-reads (BioGraph, Manta) across references. Note that chm13 results were not generated for Manta. Additionally, SURVIVOR failed to merge the Manta results. [file 13059_2022_2840_MOESM7_ESM.png]
