# Supplementary material for: Truvari: refined structural variant comparison preserves allelic diversity
Source: Genome Biol. 2022 Dec 27;23:271. doi: 10.1186/s13059-022-02840-6 (PMC9793516; doi:10.1186/s13059-022-02840-6)
Supplement: Supplementary file 17 — Additional file 17: Review history. [file 13059_2022_2840_MOESM17_ESM.docx]

**1^st^ round**

**Reviewer 1**

This manuscript proposes a toolkit, Truvari, for comparing and merging structural variants (SVs) calls from multiple samples or datasets. Truvari uses multiple metrics for evaluating the similarity between a pair of SVs, and identify a set of thresholds for the metrics for merging SVs to achieve a good balance between "under"-merging (redundant SVs exist) and "over"-merging (true SVs are missed). Although I think merging SVs across samples or dataset is an important task to facilitate downstream analysis and comparison across patients or conditions, there are many concerning points in this manuscript.

1. There are many confounders that affect the similarity between SV calls across datasets, for example, sequencing depth, methods to call SVs. However, the evaluation of Truvari doesn't consider the sequencing depth, and the metric thresholds are chosen based on only one SV caller. Without benchmarking in details with respect to these confounders, the generalizability of Truvari is concerning.

2. The benchmarking is done on real data only using mainly indirect evaluations, such as number of SVs, allele frequency. The direct evaluating using precision and recall especially for inter-sample merging is hard due to the lack of ground truth. In this case, evaluating using simulated data is helpful for reassuring the accuracy of SV merging and performance comparison against other methods.

3. It is mentioned that tandem repeats should be treated differently than other regions, where have a stringent criteria of merging tends to preserve allele specific SVs. Then why not directly incorporate tandem repeat information in designing Truvari and determining thresholds?

4. There are many unexplained intuitions as well as undefined variables in the writing.

a. "The simplest case of SV merging would be to combine SVs across halo types within a sample". Allele-specific or haplotype-specific SVs have a richer information than plain SVs. Why is it desired to combine haplotypes? Is it due to VCF format doesn't distinguish haplotypes?

b. What is allele frequency and why is it desired to have a low allele frequency?

c. What is the formula for het/hom ratio? What's the expected range of this quantity?

d. How exactly is "recall" defined in matching SVs, as well as TP and FP? And why exact merge has a low recall compared to loose merge? Conceptually, exact merge is the direct set union of SVs and should have perfect recalls.

5. There is a nice comparison with many other SV merging methods, however, there is no background or overview of the current available methods. Other methods exists than the ones in the comparison, for example:

Larson, David E., et al. "Svtools: population-scale analysis of structural variation." Bioinformatics 35.22 (2019): 4782-4787.

**Reviewer 2**

Given the present proliferation of sequencing technologies and data, in addition to challenges in establishing SV representation standards, the importance of harmonizing SV call sets cannot be understated. The authors present a novel method to address this challenge. Unlike previous approaches, their tool, Truvari, uses local sequence alignment as an additional criterion for matching variants. This approach has significant advantages in certain contexts and overcomes technical issues with SV representation, particularly in repetitive regions where precise breakpoints may not be well defined.

The manuscript presents a series of analyses of Truvari's performance on an SV call set of 36 high-quality genomes resolved from long-read sequencing.

Applying the method to haplotype pairs from individual samples (intra-sample), it is shown that the method matches (a) SVs to GIAB truth set on NA24385 and (b) SVs between haplotypes that likely correspond to homozygous sites. A sensitivity analysis of model parameters on final variant counts is performed and shows that, as expected, reducing matching stringency causes a decrease in total variant count, reflecting the continuum of precision in breakpoint calling.

The authors then present inter-sample analyses and compare results to other tools including Jasmine, SURVIVOR, and a naïve clustering implementation, with the exact-matching BCFTools results as a point of reference. They compare mean allele frequency (AF), accuracy against GIAB, and population metrics across tools. They show that Truvari generally yields lower mean AF, and that it outperforms other tools' accuracies on 216 SVs located in 273 clinically relevant CMRG genes.

Finally, a comparison of allele collapsing in repetitive regions is performed to show how Truvari's sequencing matching permits some alleles to be properly collapsed while preventing over-clustering of distinct alleles (i.e. different repeat numbers).

The presented method is a significant contribution and has potential of very high impact in the field. However, I do have some concerns about the overall narrative of the manuscript. In particular, the primary claim that Truvari yields improved allelic diversity over other tools by preventing over-clustering is not sufficiently substantiated in the current manuscript. I have suggestions for how it could be improved with additional analyses to make the authors' argument more compelling.

Major comments:

* There is a difficulty in that Truvari itself is a benchmarking tool but is used to benchmark its own performance. The authors should first establish that the tool matches variants correctly as expected on an orthogonal truth set in which true matches and non-matches are known. To this end, an in silico experiment using the "bench" and "collapse" methods on a sample against biological duplicates with simulated technical noise added would provide a compelling evidence that not only does Truvari match variants with high accuracy but also that the variants matched are the correct ones.

* On a related note, I believe that there is a missed opportunity to showcase the advantages of the sequence similarity approach. There has been a proliferation of tools in both the short- and long-read SV space, and a major practical issue is that SV representation is not well standardized. The authors focused on benefits in repetitive regions. However, there are many other cases of ambiguous representations where this is useful. For example, a tandem duplication can also be represented as an insertion located at either the 5' or 3' end of the duplicated segment (or, technically, any position in between). This presents challenges for overlap-dependent clustering methods that the presented tool elegantly overcomes. The manuscript would benefit from another simple in silico experiment in which biological replicates are generated with different but equivalent representations of variants. This would serve to highlight a significant problem that the tool solves and also to set up the current tandem repeat analysis.

* The authors need to demonstrate reasonable efforts toward adjusting the other tools' parameters (possibly in the Methods section) and show that such adjustments would not affect the overall interpretation of the comparison analyses. This is particularly important in this study, as Truvari seems to prevent the most under-clustering but also has a greater proportion of redundant alleles (Fig. 4c). It's unclear whether parameter adjustments to the other tools could balance out the under- and over-clustering to perform similarly.

* The intra-sample benchmarking section discusses a number of results exploring different parameter sets, such as their effects on overall variant count, accuracy against GIAB, het/hom ratios, and with different references. With the exception of the accuracy against GIAB, it is not clear how to interpret these results. The authors should provide more context and discussion to help the reader understand what the significance of these results are.

* The first inter-sample benchmarking section compares SV counts across tools. Again, this result would seem to depend on tool parameters. Figure 3b shows the corresponding reduction in mean AF, which mathematically must be true. This analysis could be improved by instead comparing the full AF distributions, in which any under- or over-clustering would be revealed by apparent skewing towards rare and common regimes, respectively.

* An inheritance analysis (i.e. Mendelian violation rates) using the 3 trios from this dataset would greatly strengthen the inter-sample analysis, as this is a straight-forward way to measure performance with respect to the balance of over- and under-clustering.

* Population-level metrics including HWE and excess heterozygosity are used, but the dataset is an admixture of ancestries. The authors need to show that the assumptions of these metrics still hold or to control for ancestry in their analysis.

* The tandem repeat analysis is very interesting and nicely demonstrates one of the key advantages of Truvari. However, the analysis also shows that Truvari under-clusters the most of all tools. The implications of this aren't completely clear as a reader. What are the relative benefits of over- vs under-clustering? Can Fig. 4 panels b and c be plotted on the same axis for a direct comparison? Some evidence that the "redundant" calls are in fact due to small differences (e.g. interspersed SNPs) would be helpful here.

* Can the authors please add a section on the computational performance of Truvari compared to the other algorithms, i.e. in terms of memory used and compute time? I suspect that the sequence alignment calculations can become costly particularly for large variants, and this would be an important factor to consider for running Truvari on larger populations.

* On a similar note, the "chunking" algorithm as described seems like it is sensitive to the presence of large calls. For example, a multi-megabase deletion could prevent the chunking strategy from effectively clustering groups of smaller deletions for efficient processing (since the algorithm performance is quadratic with chunk size). Can the author please clarify if this is possible? If so, I think it should be acknowledged in the discussion as a possible area for future improvements.

* The authors state that Truvari can handle basic SV types including deletions, duplications, inversions, and insertions. This should be mentioned earlier in the manuscript in addition to some justification for only including deletions and insertions in the analyses. Furthermore, the addition of inter-chromosomal events (such as reciprocal translocations) and unresolved break-ends should be mentioned as another area for future work.

* Is 0 the default threshold for reciprocal overlap in Truvari? It was a bit confusing at first as to why this was the case until carefully thinking through the tandem repeat section. Some discussion of this choice is warranted. In what use cases would it be useful to require overlap?

Minor comments:

P1-L44: "of the genome" is redundant

P2-L33: Examples of such insertions would be helpful to cite

P2-L40: "We use the experience gained…" could be replaced with "We incorporate lessons from…"

P2-L52: "pVCF" should be defined here

Figure-1: I noticed the use of "base" to refer to one of the call sets, and I think it leads to some confusing text as this word is overloaded with the "base" that refers to a nucleotide. If possible, I'd suggest a less ambiguous term like "baseline".

Figure-2a: These pair plots are a bit saturated with data points. Is there a way to illustrate point density? Also besides the sites with low reciprocal overlap but high sequence size/similarity, I'm not sure there is much critical information conveyed here.

Figure-2b: This is a fantastic use of violin plots to supplement the box plots and raw datapoints. I'd suggest making the trend line black since it looks somewhat orange to my eye and clashes with the "strict" column.

P5-L12-20: I found this section a bit wordy and difficult to follow, consider revising. The 8:1127 ratio should be reduced to 1:141 for clarity.

P5-L32-42: Specify which parameter set was used.

P6-L15: I believe this is the first time the term "locus" is referenced, and it is used elsewhere in the manuscript. It took several re-reads to understand what this referred to, please clarify here.

P8-L11-14: Incorporate a brief explanation of how "missing" and "redundant" were actually determined - i.e. by counting repeats with TRF and comparing to the reference count.

P10-L15: Clarify that you a referring to the number of unique SVs across a population (as opposed to per genome).

**Reviewer 3**

English et al. propose a new tool, named as Truvari, for the comparison of structure variants (SVs). Such tools are quite on-demand to genomic studies due to the ever-increasing samples being sequenced and there are many structure variants over-represented. The results on real datasets indicated that Truvari has the ability to merge the SVs which are potentially identical but have different representations in the callsets of various samples. The manuscript is also well-organized. However, I think that there are still a couple of issues should be addressed before publication.

1. In the benchmark, the SVs of various samples were called by the same pipeline (i.e., long-read, haplotype-resolved assemblies + Minimap2 + paftools). This is a commonly used assembly-based approach. However, what about alignment-based SV calling approach? Usually, alignment-based SV calling pipelines could have more divergent outputs, is Truvari able to well-handle such callsets? I suggest the authors to supply additional results on the callsets produced by alignment-based tools, such as Sniffles, CuteSV, SVIM, etc.

2. Moreover, the ability of Truvari to handle SV callsets from various pipelines is also not very clear. Multiple tools/pipeline are usually employed to make more confident callsets in one genomic study. What role will Truvari play in such a scenario? I also suggest the authors to supply some more benchmarks.

3. The authors mentioned in Discussion that "Truvari's flexibility allows it to be used on any VCF with SVs, even those generated by short reads." What if Truvari works on short read-based callsets as there is usually no sequence information provided? Furthermore, what if Truvari works for a hybrid callsets that some of the samples have long read-based callsets and other samples have short read-based ones? Is it useful to improve the quality of short read-based callsets with long reads?

4. It is shown in the manuscript that Truvari can compare and merge the SVs in various haplotypes of a sample. But it is not clear whether the corresponding genotypes would be revised after the processing? For example, is the heterozygous SVs being changed to homozygous?

5. The authors showed that Truvari helps to compare and merge the SVs in various copies of tandem repeats. This is very useful to downstream analysis. However, the writings about this function is not very clear for me. I hope the authors can provide more explanations. It could be better to give some examples in real data.

6. The sequence similarity is calculated based on edit distance. Why such model is used? I think the difference in the sequences of identical SVs could be caused by a number of issues. Edit distance model is straightforward, but is it a little too simple? Did you try other more advanced scoring systems?

7. I think it is better to add some performance of Truvari, such as time cost and memory footprint, as the information could be also important to users.

**Authors’ response**

We thank the reviewers and editor for their comments and help in improving this manuscript about SV comparison and Truvari. We have now incorporated suggestions and modified the manuscript to improve the presentation of our insights. We highlighted these changes in the main text and also gave point responses here to the individual suggestions and questions from the reviewers.

One of the main comments from the reviewers which we’d like to clarify is that we are not claiming to fully solve the issue of SV merging, but only to significantly improve researcher’s understanding of the problem while providing a tool to solve the most frequent general use cases. There are many challenges to merging SV in terms of highly repetitive regions where vastly different representations of SV could still impact the merging (e.g. centromeric regions, LPA etc). We think that this paper resolves the majority of issues for SV merging but also introduces Truvari and its abilities to cope with them. Truvari has been maintained for 4.5 years and we plan to continue this for the future.

Reviewer #1:

This manuscript proposes a toolkit, Truvari, for comparing and merging structural variants (SVs) calls from multiple samples or datasets. Truvari uses multiple metrics for evaluating the similarity between a pair of SVs, and identify a set of thresholds for the metrics for merging SVs to achieve a good balance between "under"-merging (redundant SVs exist) and "over"-merging (true SVs are missed). Although I think merging SVs across samples or dataset is an important task to facilitate downstream analysis and comparison across patients or conditions, there are many concerning points in this manuscript.

1. There are many confounders that affect the similarity between SV calls across datasets, for example, sequencing depth, methods to call SVs. However, the evaluation of Truvari doesn't consider the sequencing depth, and the metric thresholds are chosen based on only one SV caller. Without benchmarking in details with respect to these confounders, the generalizability of Truvari is concerning.

We thank the reviewer for this comment but we feel there has been a misunderstanding of some kind. Truvari is not a SV caller but focuses on the comparison of SV call sets. This is far from solved research area: “When are two SV the same allele?”. In contrast to SNV, SV are mainly occurring in tandem repeats and thus require more accurate merging techniques. Our manuscript in fact shows what is happening with tools that are available today in terms of undermerging (e.g. bcftools) or overmerging (e.g. SURVIVOR). Since the focus of the paper is merging and comparison of SV instead of calling SV we don’t need to take coverage into account.

2. The benchmarking is done on real data only using mainly indirect evaluations, such as number of SVs, allele frequency. The direct evaluating using precision and recall especially for inter-sample merging is hard due to the lack of ground truth. In this case, evaluating using simulated data is helpful for reassuring the accuracy of SV merging and performance comparison against other methods.

We considered simulated data, but this data would not recapitulate the complexity of SV merging/comparison. Instead, the intra-sample section includes benchmarking against the truth-set generated by the Genome In a Bottle Consortium's (GIAB) HG002 v0.6 Tier1 SVs , which is a product of the National Institute of Standards and Technology. The inter-sample section included a comparison to GIAB’s “Challenging Medically Relevant Genes” benchmark. Accurately simulating inconsistencies in SV representations would require accounting for confounding factors of sequencing errors, alignment ambiguities, reference biases, and allelic diversity. Most SV (80-90%) are occuring in tandem repeats and all SV simulation methods that we are aware of assume a random model of size, location etc. Thus, instead of relying on oversimplified simulated data we systematically show the patterns of over and under merging from tools by comparing against a high confidence benchmarking data set

3. It is mentioned that tandem repeats should be treated differently than other regions, where have a stringent criteria of merging tends to preserve allele specific SVs. Then why not directly incorporate tandem repeat information in designing Truvari and determining thresholds?

We actually don’t state that they should be treated differently, but say that they represent unique challenges in terms of variant representation. Since most SV are occurring in tandem repeats, it's important to get these right. One reason why we didn't want to fall back to an annotation file is that Truvari is not only implemented for a specific genome build but can be used throughout all species. Thus, improving the field of SV merging in general.

4. There are many unexplained intuitions as well as undefined variables in the writing.

We tried to improve this now.

a. "The simplest case of SV merging would be to combine SVs across halo types within a sample".

Allele-specific or haplotype-specific SVs have a richer information than plain SVs. Why is it desired to combine haplotypes? Is it due to VCF format doesn't distinguish haplotypes?

This might be a misunderstanding and we have now improved the main text. Our approach was to first investigate how SV merging impacts the VCF file over studying the consequences on a per haplotype level per individual. The theory here is that SV can still be over merged or under when they are compared in the simplest case of per haplotype.

b. What is allele frequency and why is it desired to have a low allele frequency?

The section was more to show that there is a significant impact of SV merging across the different strategies. Based on this demonstration of a problem, we then refine the approaches and compare them deeper. We do not state that a lower or higher allele frequency is good or bad at this point of the manuscript.

c. What is the formula for het/hom ratio? What's the expected range of this quantity?

Here we only computed the heterozygous vs. homozygous variant counts. We have now clearly defined that in the main text.

d. How exactly is "recall" defined in matching SVs, as well as TP and FP? And why exact merge has a low recall compared to loose merge? Conceptually, exact merge is the direct set union of SVs and should have perfect recalls.

We assume the reviewer is referring to the comparison of strict, exact and loose in merging strategies from 2 haplotype calls and comparison to GIAB call set. Here strict and exact are reporting 93.7% and loose 93.6%. Thus following the expectations that the exact method leads to slightly higher recall, but lower precision as undermering tends to do this. We now also added sentences in the caption of the Supplementary Table 1 to make the content of it clearer.

5. There is a nice comparison with many other SV merging methods, however, there is no background or overview of the current available methods. Other methods exists than the ones in the comparison, for example:

Larson, David E., et al. "Svtools: population-scale analysis of structural variation." Bioinformatics 35.22 (2019): 4782-4787.

We thank the reviewer for highlighting this. We have discussed a few general strategies for SV merging in the introduction and highlighted the consequences. In this version we have extended this description to make it clearer. For SVTools, this appears to be a SV calling pipeline and is not solely focused on SV merging. Their strategy of comparing breakpoints is represented by the tool SURVIVOR or the “naive” tool (i.e. 50% reciprocal overlap). Furthermore, as svtools’ github hasn’t been updated since 2019, we assumed it is no longer under active development.

Reviewer #2:

Given the present proliferation of sequencing technologies and data, in addition to challenges in establishing SV representation standards, the importance of harmonizing SV call sets cannot be understated. The authors present a novel method to address this challenge. Unlike previous approaches, their tool, Truvari, uses local sequence alignment as an additional criterion for matching variants. This approach has significant advantages in certain contexts and overcomes technical issues with SV representation, particularly in repetitive regions where precise breakpoints may not be well defined. The manuscript presents a series of analyses of Truvari's performance on an SV call set of 36 high-quality genomes resolved from long-read sequencing.

Applying the method to haplotype pairs from individual samples (intra-sample), it is shown that the method matches (a) SVs to GIAB truth set on NA24385 and (b) SVs between haplotypes that likely correspond to homozygous sites. A sensitivity analysis of model parameters on final variant counts is performed and shows that, as expected, reducing matching stringency causes a decrease in total variant count, reflecting the continuum of precision in breakpoint calling.

The authors then present inter-sample analyses and compare results to other tools including Jasmine, SURVIVOR, and a naïve clustering implementation, with the exact-matching BCFTools results as a point of reference. They compare mean allele frequency (AF), accuracy against GIAB, and population metrics across tools. They show that Truvari generally yields lower mean AF, and that it outperforms other tools' accuracies on 216 SVs located in 273 clinically relevant CMRG genes. Finally, a comparison of allele collapsing in repetitive regions is performed to show how Truvari's sequencing matching permits some alleles to be properly collapsed while preventing over-clustering of distinct alleles (i.e. different repeat numbers).

The presented method is a significant contribution and has potential of very high impact in the field. However, I do have some concerns about the overall narrative of the manuscript. In particular, the primary claim that Truvari yields improved allelic diversity over other tools by preventing over-clustering is not sufficiently substantiated in the current manuscript. I have suggestions for how it could be improved with additional analyses to make the authors' argument more compelling.

We would like to thank the reviewer for highlighting the importance of this analysis and approach.

Major comments:

1. There is a difficulty in that Truvari itself is a benchmarking tool but is used to benchmark its own performance. The authors should first establish that the tool matches variants correctly as expected on an orthogonal truth set in which true matches and non-matches are known. To this end, an in silico experiment using the "bench" and "collapse" methods on a sample against biological duplicates with simulated technical noise added would provide a compelling evidence that not only does Truvari match variants with high accuracy but also that the variants matched are the correct ones.

We thank the reviewer for this suggestion and indeed there is a risk of overestimation. We are using truvari bench when comparing to the GIAB call set following published recommendations. However, we now made this clearer that Truvari is already benchmarked for this capability and is being used for work by GIAB (PMC8454654, 150 citations). In fact, National Institute of Standards and Technologies recommends Truvari as a benchmark method. In order to reduce overestimation, we developed and described the ‘Assessing performance of merging tools’ section in which we use orthogonally derived annotations from Tandem Repeat Finder as a benchmark.

The other challenge of benchmarking is we can only simulate events for being merged once we understand the underlying mechanisms, which this work aims to explore. In our experience, tools which attempt to accurately simulate the inconsistencies of SV representations fall short. Only a few in silico approaches are even moderately more sophisticated than randomly picking reference spans. However, SVs do not occur uniformly across the genome and are enriched in regions which are highly redundant and of lower complexity. Accurately modeling the confounding factors of sequencing errors, alignment ambiguities, reference biases, and allelic diversity would be an incredible research achievement. Therefore, we leveraged real data and demonstrated how the independent step of SV merging effects discovered SV’s benchmarking against well established truth-sets in order to avoid inaccurate modeling while still confronting the difficulties encountered during SV evaluation.

2. On a related note, I believe that there is a missed opportunity to showcase the advantages of the sequence similarity approach. There has been a proliferation of tools in both the short- and long-read SV space, and a major practical issue is that SV representation is not well standardized. The authors focused on benefits in repetitive regions. However, there are many other cases of ambiguous representations where this is useful. For example, a tandem duplication can also be represented as an insertion located at either the 5' or 3' end of the duplicated segment (or, technically, any position in between). This presents challenges for overlap-dependent clustering methods that the presented tool elegantly overcomes. The manuscript would benefit from another simple in silico experiment in which biological replicates are generated with different but equivalent representations of variants. This would serve to highlight a significant problem that the tool solves and also to set up the current tandem repeat analysis.

We thank the reviewer for their suggestion and we updated the text appropriately. For the above mentioned challenges for simulations it's hard to accurately represent this. Nevertheless, we think this is also partially highlighted by the population merge where common SV are shared but potentially wrongly merged with rare SV depending on the SV merging strategy.

3. The authors need to demonstrate reasonable efforts toward adjusting the other tools' parameters (possibly in the Methods section) and show that such adjustments would not affect the overall interpretation of the comparison analyses. This is particularly important in this study, as Truvari seems to prevent the most under-clustering but also has a greater proportion of redundant alleles (Fig. 4c). It's unclear whether parameter adjustments to the other tools could balance out the under- and over-clustering to perform similarly.

This is an interesting suggestion but would put us at our limitations. These SV merging methods have all clearly recommended parameters.

The choice of comparison tools is in and of itself a parameter sweep of the larger SV comparison space since they use overlapping similarity metrics (e.g. all of them use a reference distance), usually with different defaults. The methods section states the default parameters used and in supplementary table 2 we provide descriptions of the metrics used by each tool as well as an ‘estimated stringency’ of their defaults. Further refinement (e.g. Moderate stringency - but a little bit tighter vs Moderate stringency - but a little bit looser) would fail to capture a reader’s attention due to the subtleties of the differences at genome scale. Parameters for merging stringency and their resultant performance is effectively a continuous distribution both within truvari and between the selected tools. We have chosen three discrete points for intra-sample merging evaluation and 5 for inter-sample merging, which we feel is an adequate parameter sweep.

To provide context, we’d like to draw attention to the Jasmine paper describing a single parameter ‘min_dist’, the Survivor paper making no mention of parameters, Bcftools having no parameters, and Naive50% reciprocal overlap having a single epemenous parameter. While newer versions of Jasmine seem to have more parameters available and Survivor has parameters available, the parameter space is enormous and doing a more detailed sweep of said space would require an enormous collection of results to pore over since each adjustment would simultaneously gain and lose true and false matches.

Instead, we use as input a ‘best case’ scenario of SVs from a haplotype-resolved, long-read assembly and report the tools’ baseline performance. This allows equality in the evaluations and provides readers with reasonable starting expectations of the tools from which they can work to maximize performance from their calls. Given that the Truvari defaults are demonstrated to perform better than the other tools’ defaults, we believe this effectively illustrates to readers that by using Truvari their first result is likely closer to the ‘perfect’ results than what they’ll encounter with other tools. This should reduce the number of attempts one needs to get everything just right, which in this context is nearly equivalent to p-hacking.

We have added text to clarify these points.

4. The intra-sample benchmarking section discusses a number of results exploring different parameter sets, such as their effects on overall variant count, accuracy against GIAB, het/hom ratios, and with different references. With the exception of the accuracy against GIAB, it is not clear how to interpret these results.

The authors should provide more context and discussion to help the reader understand what the significance of these results are.

We extended the main text to better describe this approach and also the discussion to highlight the differences that are observed and the impact of them.

5. The first inter-sample benchmarking section compares SV counts across tools. Again, this result would seem to depend on tool parameters. Figure 3b shows the corresponding reduction in mean AF, which mathematically must be true. This analysis could be improved by instead comparing the full AF distributions, in which any under- or over-clustering would be revealed by apparent skewing towards rare and common regimes, respectively.

This is an interesting thought, but if we understood the reviewer correctly, their proposal is to compare against a ‘true’ allele frequency distribution measured from a perfectly merged pVCF. This baseline, however, does not exist. Without it, checking for skews would require an assumption that the reference always holds the universal major allele. However, this is not a valid assumption as ~70% of GRCh38 is sourced from a single individual and 100% of chm13. Therefore, it’s unclear how to interpret skews towards rare variants as undermering instead of the major allele in the reference and skews towards common variants as being over merging and not a minor allele in the reference. Instead, we use the orthogonally derived annotations from Tandem Repeat Finder as described in the section ‘Assessing performance of merging tools‘ to quantify the merging accuracy while sections using the more relativistic AF highlight the impact of SV merging strategies given identical inputs.

6. An inheritance analysis (i.e. Mendelian violation rates) using the 3 trios from this dataset would greatly strengthen the inter-sample analysis, as this is a straight-forward way to measure performance with respect to the balance of over- and under-clustering.

Mendelian error rates are an excellent quality control metric. However, we do not believe it is useful for measuring performance. Specifically, we’ve reasoned that in situations where it’s possible to find matching calls across all haplotypes - thus creating an ‘all homozygous alternate’ allele in the pVCF - there would not be a mendelian violation. These ‘all hom alts’ can arise due to being truly matching variants or from false matches (i.e. over-merging). Therefore, mendelian error rates cannot distinguish correct-merging from over-merging. We believe this to be an unbalanced metric which should not be relied upon as an interpretation of performance and won’t be presenting the results in the manuscript in order to avoid misleading readers.

In the interest of transparency, we present to the reviewer the results of the mendelian error analysis which was already performed and available via the paper’s github. First, the left figure shows the mendelian error rates of the inter-sample merges across all SVs and references. The right figure shows the mendelian error rates when only considering variants present (i.e. non-reference homozygous) in at least one individual of a trio.

7. Population-level metrics including HWE and excess heterozygosity are used, but the dataset is an admixture of ancestries. The authors need to show that the assumptions of these metrics still hold or to control for ancestry in their analysis.

Here we are using HWE as a comparison method between different merging approaches based on the same input SV call set. Thus showing an impact of HWE not based on ethnicities or other external parameters. If all methods were to perform equally we would expect the HWE to be the same, but clearly they are not.

8. The tandem repeat analysis is very interesting and nicely demonstrates one of the key advantages of Truvari. However, the analysis also shows that Truvari under-clusters the most of all tools. The implications of this aren't completely clear as a reader. What are the relative benefits of over- vs under-clustering? Can Fig. 4 panels b and c be plotted on the same axis for a direct comparison? Some evidence that the "redundant" calls are in fact due to small differences (e.g. interspersed SNPs) would be helpful here.

We have tried to better highlight the paragraph in the discussion which attempts to address this point of truvari under-merging. For figure 4, we understand the reviewer’s confusion. We made a mistake as the panel labeling is misleading. Figure 4b is “Missing variants per-locus” and we have relabeled the x-axis as ‘Variant Count”. Figure 4c is now titled “Number of Loci with Redundant calls” and the x-axis relabeled to “Variant Count”. Please let us know if this clarifies the figure.

9. Can the authors please add a section on the computational performance of Truvari compared to the other algorithms, i.e. in terms of memory used and compute time? I suspect that the sequence alignment calculations can become costly particularly for large variants, and this would be an important factor to consider for running Truvari on larger populations.

This publication was written in conjunction with Truvari version 3.1 which was the last release before we chose to focus on optimization. As early as version 3.2 we were able to implement a very simple change to use a 10x faster operation. In version 4.0-dev, we’re seeing an overall runtime that’s over 2x faster than 3.1 and that’s with other changes that will affect performance still pending. Regardless, since multiple reviewers were interested in this, we have now included a small section with the outdated runtimes.

10. On a similar note, the "chunking" algorithm as described seems like it is sensitive to the presence of large calls. For example, a multi-megabase deletion could prevent the chunking strategy from effectively clustering groups of smaller deletions for efficient processing (since the algorithm performance is quadratic with chunk size). Can the author please clarify if this is possible? If so, I think it should be acknowledged in the discussion as a possible area for future improvements.

Every call in a cluster is compared to every call in a cluster. Chunking with large variants may make a lot of comparisons that evaluate to false, but the true comparisons are still made. As mentioned above, we’re still actively maintaining and improving Truvari, including the capability to compare variants under 50bp. As one can imagine, there are many more variants under 50bp which clearly calls into question the all against all chunking strategy and is being addressed. At the same time, I would like to draw attention to why the all vs all chunking comparison was implemented. By comparing all calls in a chunk against one another, we’re able to exhaustively annotate FP/FN with information about the similarity of a call’s closest match. This helps users with their downstream analysis. For example, a user may start a benchmarking analysis with default thresholds and find lower than expected performance. Instead of needing to run truvari several times, they can take advantage of tools like `truvari vcf2df` as well as pandas and seaborn to perform exploratory data analysis and find exactly how and why their calls are matching. Any short-circuiting of the chunking needs to responsibly account for this reported information and is unfortunately not as simple as something like, “chunk calls over 10kb separately from those under 10kb” as these types of thresholds create edge conditions. As you can see, we could talk at length about the usability design of the tool and the balance of optimizations and accuracy. However, we feel that the publication space is premium and should be dedicated to providing a suscient explanation of the more fundamental insights we aim to provide about SV comparison without bloviating as we have done here.

11. The authors state that Truvari can handle basic SV types including deletions, duplications, inversions, and insertions. This should be mentioned earlier in the manuscript in addition to some justification for only including deletions and insertions in the analyses. Furthermore, the addition of inter-chromosomal events (such as reciprocal translocations) and unresolved break-ends should be mentioned as another area for future work.

We have included this now earlier in the result section. Fundamentally, every SV can be represented as some combination of Insertion and Deletion events (e.g. an inversion is a deletion of the reference sequence and insertion of the reverse complement sequence). Therefore, we focused on these SV types in order to introduce the concepts of SV comparison. Solving for every possible variant representation and their combinations is outside of the scope of this research.

12. Is 0 the default threshold for reciprocal overlap in Truvari? It was a bit confusing at first as to why this was the case until carefully thinking through the tandem repeat section. Some discussion of this choice is warranted. In what use cases would it be useful to require overlap?

Yes. And it’s actually not just tandem repeats where this is the consideration. We state in the background that “...reciprocal-overlap is not applicable to sequence-resolved insertions, which have no physical span over the reference.” We clarified the text appropriately.

Minor comments:

These have been addressed in the main text.

P1-L44: "of the genome" is redundant

P2-L33: Examples of such insertions would be helpful to cite

P2-L40: "We use the experience gained..." could be replaced with "We incorporate lessons from..."

P2-L52: "pVCF" should be defined here

Figure-1: I noticed the use of "base" to refer to one of the call sets, and I think it leads to some confusing text as this word is overloaded with the "base" that refers to a nucleotide. If possible, I'd suggest a less ambiguous term like "baseline".

Figure-2a: These pair plots are a bit saturated with data points. Is there a way to illustrate point density?

Also besides the sites with low reciprocal overlap but high sequence size/similarity, I'm not sure there is much critical information conveyed here.

Figure-2b: This is a fantastic use of violin plots to supplement the box plots and raw datapoints. I'd suggest making the trend line black since it looks somewhat orange to my eye and clashes with the "strict" column.

P5-L12-20: I found this section a bit wordy and difficult to follow, consider revising. The 8:1127 ratio should be reduced to 1:141 for clarity.

P5-L32-42: Specify which parameter set was used.

P6-L15: I believe this is the first time the term "locus" is referenced, and it is used elsewhere in the manuscript. It took several re-reads to understand what this referred to, please clarify here.

P8-L11-14: Incorporate a brief explanation of how "missing" and "redundant" were actually determined - i.e. by counting repeats with TRF and comparing to the reference count.

P10-L15: Clarify that you a referring to the number of unique SVs across a population (as opposed to per genome).

Reviewer #3:

English et al. propose a new tool, named as Truvari, for the comparison of structure variants (SVs). Such tools are quite on-demand to genomic studies due to the ever-increasing samples being sequenced and there are many structure variants over-represented. The results on real datasets indicated that Truvari has the ability to merge the SVs which are potentially identical but have different representations in the callsets of various samples. The manuscript is also well-organized. However, I think that there are still a couple of issues should be addressed before publication.

1. In the benchmark, the SVs of various samples were called by the same pipeline (i.e., long-read, haplotype-resolved assemblies + Minimap2 + paftools). This is a commonly used assembly-based approach. However, what about alignment-based SV calling approach? Usually, alignment-based SV calling pipelines could have more divergent outputs, is Truvari able to well-handle such callsets? I suggest the authors to supply additional results on the callsets produced by alignment-based tools, such as Sniffles, CuteSV, SVIM, etc.

We used assembly based approaches here to obtain consistent and best possible representation across the entire genome. Truvari is capable of running and coping with different read based methods and is thus recommended by GIBA (NIST) to benchmark SVs across short or long reads. Please also see belows response as we think that point 1 & 2 are related.

2. Moreover, the ability of Truvari to handle SV callsets from various pipelines is also not very clear. Multiple tools/pipeline are usually employed to make more confident callsets in one genomic study. What role will Truvari play in such a scenario? I also suggest the authors to supply some more benchmarks.

This paper is focusing on the best case scenario for assembly based calling and to derive lessons learned from such comparison and motivate the implementation of them inside of Truvari. This is indeed an interesting idea to discuss multi-technology calling but we feel is out of scope. The multi technology calling is highly dependent on what technologies are available and their individual properties. For example, while HiC can be used to call SV their breakpoints are often inaccurate, similar to e.g. bionano. The latter has even bigger challenges as SV are only observed indirectly and thus no sequence information will be available. For ONT one has often discussed limitations for smaller deletions and for array technologies further complications with breakpoints and DUP vs. DELs. This makes the request from the reviewer untraceable. Nevertheless, we included a paragraph now in the discussion to make the reader aware of this.

3. The authors mentioned in Discussion that "Truvari's flexibility allows it to be used on any VCF with SVs, even those generated by short reads." What if Truvari works on short read-based callsets as there is usually no sequence information provided? Furthermore, what if Truvari works for a hybrid callsets that some of the samples have long read-based callsets and other samples have short read-based ones? Is it useful to improve the quality of short read-based callsets with long reads?

Truvari is able to cope with these challenges. However, as you can imagine not having the sequence information available reduces the ability to correctly merge SV in repetitive context or other challenges. We’ve updated the text to more clearly explain this. To demonstrate Truvari’s flexibility, as well as address reviewer’s point #7, we have added a section to the results titled “Computational Performance”

4. It is shown in the manuscript that Truvari can compare and merge the SVs in various haplotypes of a sample. But it is not clear whether the corresponding genotypes would be revised after the processing? For example, is the heterozygous SVs being changed to homozygous?

The short answer is that yes truvari does this. This was mentioned in the methods which have been clarified and we further added information on this in the main text as well. This feature’s effect is illustrated by the het/hom ratio comparison.

5. The authors showed that Truvari helps to compare and merge the SVs in various copies of tandem repeats. This is very useful to downstream analysis. However, the writings about this function is not very clear for me. I hope the authors can provide more explanations. It could be better to give some examples in real data.

We agree that this work does set up useful analysis of tandem repeats. T A thorough exploration of tandem repeats - a subset of SVs - requires coverage of many concepts which we feel may distract readers from the core generalized concepts of SV comparison addressed in this work. We’ve updated the text to try and better strike the balance between exploring tandem repeats in great depth and focusing on describing the challenges of SV comparison genome wide.

6. The sequence similarity is calculated based on edit distance. Why such model is used? I think the difference in the sequences of identical SVs could be caused by a number of issues. Edit distance model is straightforward, but is it a little too simple? Did you try other more advanced scoring systems?

Our assessments showcase that sequence similarity is one of the key metrics to define if two SV are the same allele or not and should thus be merged. This is for the first time highlighted in Figure 2A. Here we compute edit distance with a pairwise alignment process thus is a comprehensive methodology. We use the edlib (PMID: 28453688) which has been often cited.

7. I think it is better to add some performance of Truvari, such as time cost and memory footprint, as the information could be also important to users.

This publication was written in conjunction with Truvari version 3.1 which was the last release before we chose to focus on optimization. As early as version 3.2 we were able to find and implement a very simple change to use a 10x faster operation. In version 4.0-dev, we’re seeing an overall runtime that’s over 2x faster than 3.1 and that’s with other changes that will affect performance still pending. Regardless, since multiple reviewers were interested in this, we have now included a small section with the outdated runtimes.

**2^nd^ round**

**Reviewer 1**

The updated manuscript addressed most of my previous comments. However, I have some further suggestions.

1. My generalizability concern hasn’t been fully addressed. The section “Matching TVs between haplotypes” established that “Strict merge” maintains a good balance of removing redundancy and avoiding missing SVs. However, in a new cohort or new dataset, it is not clear whether the “Strict merge” is still recommended. Or if not, how to set the thresholds.

2. SV here seems to mainly refer to deletion and tandem duplication, and inversion and translocations are ignored. But should be made clear of exactly what types of SV are considered.

3. For the bar-chart of the number of SVs of each type, it would be helpful to include the counts both before merging.

4. The overview figure of Truvari only includes the methodology part, but it would be more comprehensive if input and output, especially different modes of Truvari (bench, collapse, annotation), are included in the overview figure.

5. The principle of HWE and ExcHet is too specific to population genetics rather than general math metrics, and thus it should be better explained here for their definitions and assumptions.

6. In the “Benchmarking pVCFs” part, exact merge results from BCFtools are used to evaluate other methods. According to this part, BCFtools is expected to have has high sensitivity but does not have a good performance in removing redundant SVs. However, Table 1 doesn’t have a proper metric to evaluate redundancy-removing performance, which is misleading in the way that BCFtools is almost the best method.

7. The sentence is hard to parse: “The ratio of how many true-positive (TP) GIAB SVs are lost and how many potentially redundant calls are removed compared to the Exact merge is 1:790 for Strict and 1:141 for Loose”. There are 6 numbers, TP and FP for 3 merging methods, but it's it’s unclear from the phrasing how the ratios are computed.

**Reviewer 2**

I’d like to thank the authors for their thoughtful responses to my comments. While they did not incorporate all my suggestions, most were intended as additions to strengthen or augment the study rather than necessities and their corresponding edits have greatly improved the manuscript.

There is one minor point I would like the authors to still address:

> 7. Population-level metrics including HWE and excess heterozygosity are used, but the dataset is an admixture of ancestries. The authors need to show that the assumptions of these metrics still hold or to control for ancestry in their analysis.

> Here we are using HWE as a comparison method between different merging approaches based on the same input SV call set. Thus showing an impact of HWE not based on ethnicities or other external parameters. If all methods were to perform equally we would expect the HWE to be the same, but clearly they are not.

I may not have been clear in the initial comment about this. The manuscript states that fewer violating calls indicate a higher quality merge. However, the dataset being used is an admixture which breaks the well-mixed population assumption of both HWE and excess heterozygosity, and so it is not clear how accurately they reflect merging quality.

For example, one could imagine a scenario where a particular ancestry is highly homozygous for a particular variant that is not present in the other subpopulations. In this case, the variant would appear to be violating HWE and a tool that changes correct hom-var to het or hom-ref, while reducing HWE violations, would in fact be incorrect. Is there any evidence, perhaps from SNV calls, that HWE assumptions hold reasonably well in this dataset that the authors can cite?

**Reviewer 3**

The revised maunuscript addresses my concerns, so that I have no more major comment. I think the manuscript meets the quality standard of Genome Biology.

**Authors’ response**

**Reviewer #1**

 The updated manuscript addressed most of my previous comments. However, I have some further suggestions.

1. My generalizability concern hasn’t been fully addressed. The section “Matching TVs between haplotypes” established that “Strict merge” maintains a good balance of removing redundancy and avoiding missing SVs. However, in a new cohort or new dataset, it is not clear whether the “Strict merge” is still recommended. Or if not, how to set the thresholds.

In performing the computational performance experiments, we used short-read sequencing sourced from the same individuals used which have long-read assemblies and two separate short-read SV callers. The resulting variants were merged with each of the tested callers and results presented in Fig S7. This figure shows that the default “Strict” parameters of Truvari collapse are generalizable across SV results from multiple tools and sequencing technologies.

2. SV here seems to mainly refer to deletion and tandem duplication, and inversion and translocations are ignored. But should be made clear of exactly what types of SV are considered.

We had highlighted in the previous submission: “While Truvari can process any SV type except unresolved breakends (BNDs), we focus here on only insertions and deletion.”  and further “ It’s important to note that Truvari is currently most useful for ‘resolved’ SVs (i.e. DEL, INS, INV, and DUP). What we have not addressed in this manuscript are the challenges of multi-technology or unharmonized pipeline based SV comparison.”  We think this addressed these concerns.

3. For the bar-chart of the number of SVs of each type, it would be helpful to include the counts both before merging.

We have updated the text to include the number of SVs having overlaps with genes.

4. The overview figure of Truvari only includes the methodology part, but it would be more comprehensive if input and output, especially different modes of Truvari (bench, collapse, annotation), are included in the overview figure.

The manuscript is focused on the merging of SV and the comparison. Thus the figure only focuses on this aspect of Truvari. The other (bench, collapse, annotation) do not rise to a significance that we are describing in detail. We have updated the text to include links to the user documentation on the github for these functionalities.

5. The principle of HWE and ExcHet is too specific to population genetics rather than general math metrics, and thus it should be better explained here for their definitions and assumptions.

We have updated the text to include more details.

6. In the “Benchmarking pVCFs” part, exact merge results from BCFtools are used to evaluate other methods. According to this part, BCFtools is expected to have has high sensitivity but does not have a good performance in removing redundant SVs. However, Table 1 doesn’t have a proper metric to evaluate redundancy-removing performance, which is misleading in the way that BCFtools is almost the best method.

Figure 3 and Figure 4 highlight the redundancy of bcftools. Table 1 shows the benchmark consequences when tools are over merging and thus produce lower performing results. While the bcftools and Truvari merge shows a high accuracy in Table 1. This is also because Table1 is based on a ~200 SV benchmark from GIAB.

7. The sentence is hard to parse: “The ratio of how many true-positive (TP) GIAB SVs are lost and how many potentially redundant calls are removed compared to the Exact merge is 1:790 for Strict and 1:141 for Loose”. There are 6 numbers, TP and FP for 3 merging methods, but it's it’s unclear from the phrasing how the ratios are computed.

We have updated the text for clarity.

**Reviewer #2:**

I’d like to thank the authors for their thoughtful responses to my comments. While they did not incorporate all my suggestions, most were intended as additions to strengthen or augment the study rather than necessities and their corresponding edits have greatly improved the manuscript.

There is one minor point I would like the authors to still address:

> 7. Population-level metrics including HWE and excess heterozygosity are used, but the dataset is an admixture of ancestries. The authors need to show that the assumptions of these metrics still hold or to control for ancestry in their analysis.

> Here we are using HWE as a comparison method between different merging approaches based on the same input SV call set. Thus showing an impact of HWE not based on ethnicities or other external parameters. If all methods were to perform equally we would expect the HWE to be the same, but clearly they are not.

I may not have been clear in the initial comment about this. The manuscript states that fewer violating calls indicate a higher quality merge. However, the dataset being used is an admixture which breaks the well-mixed population assumption of both HWE and excess heterozygosity, and so it is not clear how accurately they reflect merging quality.

For example, one could imagine a scenario where a particular ancestry is highly homozygous for a particular variant that is not present in the other subpopulations. In this case, the variant would appear to be violating HWE and a tool that changes correct hom-var to het or hom-ref, while reducing HWE violations, would in fact be incorrect. Is there any evidence, perhaps from SNV calls, that HWE assumptions hold reasonably well in this dataset that the authors can cite?

We thank the reviewer for their assistance in improving our manuscript. We appreciate this concern from the reviewer, and we broadly agree that care must be taken when interpreting the results of Hardy-Weinberg tests on diverse and structured populations. However, we still believe that the tests are useful for flagging potential spurious genotypes, as implemented in genotyping best practice pipelines (e.g., GATK) as a standard method of quality control.

As noted in the hypothetical example provided by the reviewer, population structure tends to increase homozygosity—a phenomenon termed the "Wahlund effect”. However, allele frequency differences among human populations tend to be subtle. A plot (attached) of allele vs. genotype frequencies of randomly selected SNPs computed on the entire diverse cohort of 1000 Genomes samples demonstrates close adherence to Hardy Weinberg expectations (dotted lines), with a slight Wahlund effect and several clear outliers that are likely best explained as genotyping errors.

Meanwhile, admixture between previously separated populations may temporarily generate excess heterozygosity, but this signature quickly erodes in the following generation. Notably, our sample is almost entirely composed of individuals from the 1000 Genomes Project, who were specifically selected on the basis that all four grandparents came from the same location or ethnic group—mitigating the impact of very recent admixture. https://www.internationalgenome.org/sites/1000genomes.org/files/docs/Informed%20Consent%20Form%20Template.pdf

In light of these points, the threshold for calling a variant as “ExcHet” is likely slightly over-conservative, as the general effect of population structure in this sample is to decrease rather than increase heterozygosity compared to Hardy-Weinberg expectations. Meanwhile, the two-tailed Hardy Weinberg test (HWE) is likely slightly, but not strongly, under-conservative. In either case, for a sample of this size (n = 38), only very strong deviations achieve the threshold of statistical significance and are much more likely to be driven by technical as opposed to demographic factors.

We now briefly note these caveats of interpretation in the Results section when these Hardy-Weinberg metrics are first introduced.

In case Genome Biology’s reviewer response system allows images, we have uploaded the figure related to the above text to our paper’s repository https://github.com/ACEnglish/TruvariData/tree/develop/manuscript. Note that in that same link we have also added figures relevant to the mendelian consistency discussion from the previous round of reviews.
